# Identification of plasma miR-4505, miR-4743-5p and miR-4750-3p as novel diagnostic biomarkers for coronary artery disease in patients with type 2 diabetes mellitus: a case-control study

**DOI:** 10.1186/s12933-024-02374-0

**Published:** 2024-07-29

**Authors:** Joanna Szydełko, Marcin Czop, Alicja Petniak, Monika Lenart-Lipińska, Janusz Kocki, Tomasz Zapolski, Beata Matyjaszek-Matuszek

**Affiliations:** 1 Department of Endocrinology, Diabetology and Metabolic Diseases, Medical University of Lublin, Jaczewskiego 8, 20-090 Lublin, Poland; 2 Department of Clinical Genetics, Medical University of Lublin, Radziwillowska 11, 20-080 Lublin, Poland; 3 Department of Cardiology, Medical University of Lublin, Jaczewskiego 8, 20-090 Lublin, Poland

**Keywords:** miRNA, Type 2 diabetes mellitus, Coronary artery disease, miRNA expression profiling, Biomarker

## Abstract

**Background:**

Type 2 diabetes mellitus (T2DM) and coronary artery disease (CAD) are commonly coexisting clinical entities with still growing incidence worldwide. Recently, circulating microRNAs (miRNAs) have emerged as novel molecular players in cardiometabolic diseases. This study aimed to identify a specific miRNA signature as a candidate biomarker for CAD in T2DM and to delineate potential miRNA-dependent mechanisms contributing to diabetic atherosclerosis.

**Methods:**

A total of 38 plasma samples from T2DM patients with and without CAD, CAD patients and healthy controls were collected for expression profiling of 2,578 miRNAs using microarrays. To investigate the regulatory role of differentially expressed (DE)-miRNA target genes, functional annotation and pathway enrichment analyses were performed utilizing multiple bioinformatics tools. Then, protein-protein interaction networks were established leveraging the STRING database in Cytoscape software, followed by cluster analysis and hub gene identification. Reverse transcription quantitative real-time polymerase chain reaction (RT-qPCR) was carried out for microarray data validation in the larger replication cohort of 94 participants. Receiver operating characteristic analysis was applied to evaluate the diagnostic values of miRNAs. Multivariate logistic regression analysis was used to develop miRNA-based diagnostic models.

**Results:**

In the discovery stage, overexpression of hsa-miR-4505, hsa-miR-4743-5p, hsa-miR-6846-5p, and down-regulation of hsa-miR-3613-3p, hsa-miR-4668-5p, hsa-miR-4706, hsa-miR-6511b-5p, hsa-miR-6750-5p, hsa-miR-4750-3p, hsa-miR-320e, hsa-miR-4717-3p, hsa-miR-7850-5p were detected in T2DM-CAD patients. The DE-miRNA target genes were significantly enriched in calcium ion binding, regulation of actin cytoskeleton, and gene expression. hsa-miR-4505, hsa-miR-4743-5p, and hsa-miR-4750-3p were found to be involved in fatty acid metabolism, leukocyte transendothelial migration, and neurotrophin signaling pathway. Dysregulation of hsa-miR-4505, hsa-miR-4743-5p, and hsa-miR-4750-3p in T2DM-CAD patients compared with T2DM subjects and controls (all *p* < 0.001) was further confirmed by RT-qPCR. All validated miRNAs demonstrated good discriminatory values for T2DM-CAD (AUC = 0.833–0.876). The best performance in detecting CAD in T2DM was achieved for a combination of three miRNAs (AUC = 0.959, 100% sensitivity, 86.67% specificity).

**Conclusions:**

Our study revealed a unique profile of plasma-derived miRNAs in T2DM patients with CAD. Potential miRNA-regulated pathways were also identified, exploring the underlying pathogenesis of CAD in T2DM. We developed a specific three-miRNA panel of hsa-miR-4505, hsa-miR-4743-5p and hsa-miR-4750-3p, that could serve as a novel non-invasive biomarker for CAD in patients with T2DM.

**Supplementary Information:**

The online version contains supplementary material available at 10.1186/s12933-024-02374-0.

## Background

Type 2 diabetes mellitus (T2DM) is a chronic metabolic disease characterized by insulin resistance and hyperglycemia, which play a crucial role in accelerating endothelial damage and the onset of cardiovascular complications, making individuals with diabetes two- to four-times more likely to develop coronary artery disease (CAD) than those without diabetes [1]. Additionally, the mortality of patients with T2DM is considerably higher in the presence of cardiovascular diseases, accounting for more than 50% of all deaths in this population [2]. The phenomenon of CAD in T2DM is multifactorial and involves an interplay of genetic, epigenetic and environmental factors, but only 25% of them have been identified so far [3, 4]. It is well known that T2DM-associated CAD has an earlier onset, faster progression, and greater extent compared to non-diabetic subjects [5]. What is more, approximately one-third of asymptomatic individuals with T2DM were found to have significant CAD with a subsequent increased risk of major adverse cardiac events [6]. Hence, early detection of CAD in patients with T2DM is becoming an important public health concern.

Currently, invasive coronary angiography remains the gold standard in the diagnosis of CAD, but it is reserved for patients whose clinical risk is assessed as high or when stress testing indicates significant ischemic burden [7, 8]. Moreover, the routine use of this technique is limited due to its invasiveness and potential side effects of ionizing radiation, contrast agent, which may generate various procedure-related and patient-dependent complications [9, 10]. Therefore, it is highly necessary to find alternative non-invasive tools that can provide additive predictive information to improve risk stratification of CAD in patients with T2DM, thus avoiding unnecessary invasive testing and actively preventing the development of diabetic atherosclerosis.

MicroRNAs (miRNAs, miRs) are a family of small (18–25 nucleotides) non-coding RNA molecules that modulate gene expression at the post-transcriptional level by inhibiting the translation of target mRNAs or inducing mRNA degradation [11]. Each specific miRNA has the ability to target multiple transcripts, while a single mRNA can be regulated by a set of miRNAs, allowing precise control of a wide range of biological processes [11]. That is why, the establishment of a specific miRNA panel may better reflect the dynamic changes of these molecules in response to disease states than a single miRNA [12]. As the number of miRNAs continues to increase, simultaneous expression profiling of hundreds of miRNAs in a single experiment appears to be indispensable to evaluate novel miRNA-mRNA interactions and clinical applications of miRNAs, particularly those not previously screened for disease [12]. Besides, it enables to avoid miRNA pre-selection bias based solely on literature overview [12]. In contrast to other RNAs, miRNAs have proven to be extremely stable in circulation and resistant to enzymatic degradation by RNase and deleterious external conditions [13]. miRNAs are also readily detectable in a variety of body fluids, including serum and plasma, highlighting their potential role as disease biomarkers [13].

Since bi-directional links between T2DM and CAD exist, the discovery of miRNAs as essential epigenetic regulators of common pathological events in cardiovascular and metabolic diseases have attracted a tremendous interest [14]. Altered expression of endothelial-specific miR-92a, miR-126, miR-210 along with miR-342 and miR-450 is known to promote hyperglycemia-induced oxidative stress and inflammation accelerating endothelial dysfunction [15–18]. Moreover, miRNAs have been reported to trigger critical processes involved in diabetic atherosclerosis, including monocyte activation, leukocyte transendothelial migration, vascular smooth muscle cell (VSMC) proliferation/migration, and platelet reactivity [14, 19]. Circulating miRNAs may strictly correspond to tissue injury, which raises endothelial-enriched miR-17, miR-92a, miR-126, cardiac-specific miR-1, miR-133, miR-499, and VSMC-enriched miR-145 as fingerprints for diabetic atherosclerosis [14]. Although, the diverse role of miRNAs in the etiology of T2DM or CAD has already been extensively explored, studies evaluating the utility of miRNA-based biomarkers in coexisting CAD and T2DM are scarce [14].

The present study aimed to investigate the differential expression of circulating miRNAs in plasma samples from T2DM patients with and without CAD, and to identify a specific miRNA signature that could serve as a potential biomarker for CAD in T2DM. In addition, target annotation and functional enrichment analyses were performed to delineate potential miRNA-dependent mechanisms contributing to the development of CAD in T2DM individuals.

## Methods

### Study design and patients

**Fig. 1:**
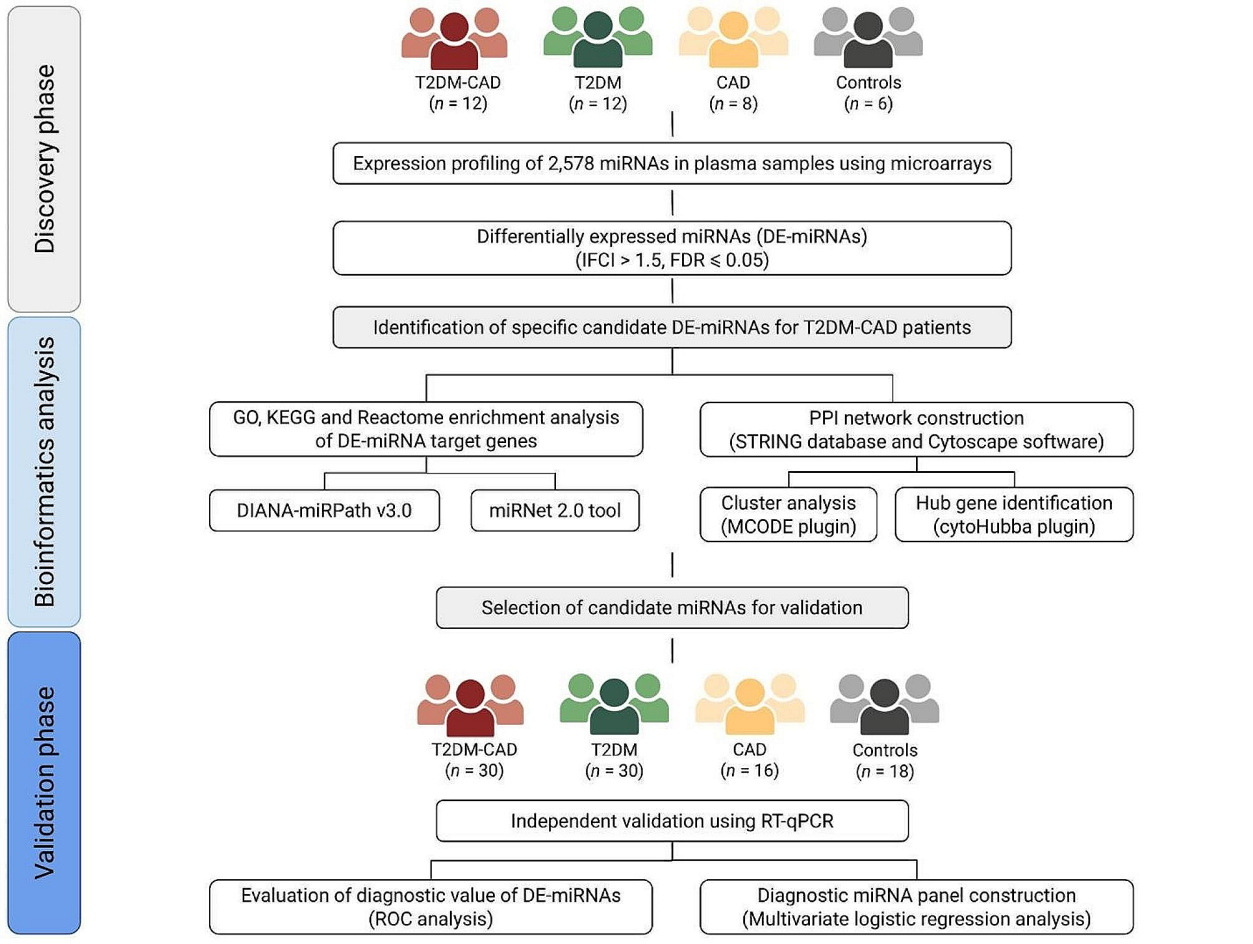
Flow chart of the study. T2DM-CAD, type 2 diabetes mellitus with coronary artery disease; T2DM, type 2 diabetes mellitus; CAD, coronary artery disease; DE-miRNA, differentially expressed miRNA; FC, fold change; FDR, false discovery rate; GO, Gene Ontology; KEGG, Kyoto Encyclopedia of Genes and Genomes; DIANA-miRPath, DNA Intelligent Analysis-miRPath; PPI, protein-protein interaction; STRING, Search Tool for the Retrieval of Interacting Genes; MCODE, Molecular Complex Detection; RT-qPCR, reverse transcription quantitative real-time polymerase chain reaction; ROC, receiver operating characteristic

This study was designed as an observational, case-control study using a two-stage approach, including primary screening and ensuing validation stage. All recruited participants were Caucasian individuals hospitalized in the Department of Endocrinology, Diabetology and Metabolic Diseases at the Medical University of Lublin, Poland, between October 2020 and December 2022. In the discovery phase, 38 patients (T2DM with CAD group, *n* = 12; T2DM group, *n* = 12; CAD group, *n* = 8; control group, *n* = 6), matched for age, sex, and body mass index (BMI), were selected for the miRNA expression profiling with microarrays. Candidate miRNAs were subsequently validated in the independent replication cohort of 94 age-, sex-, and BMI-matched patients (T2DM with CAD group, *n* = 30; T2DM group, *n* = 30; CAD group, *n* = 16; control group, *n* = 18) using reverse transcription quantitative real-time polymerase chain reaction (RT-qPCR). The overall design and key stages of the study are outlined in the flow chart (Fig. 1).

The diagnosis of T2DM was established according to the American Diabetes Association criteria [20]. Patients with T2DM and/or CAD underwent invasive coronary angiography which was assessed by two independent interventional cardiologists, and CAD was defined as at least one major epicardial vessel with > 50% stenosis, while those without any stenosis or luminal irregularities in any of the epicardial arteries were classified as CAD negative ones [21]. The control subjects were randomly selected from healthy volunteers who had normal glucose tolerance and no evidence of CAD.

Detailed information about demographic data, medical history, current medication, alcohol consumption, and smoking habits were obtained from each participant. The study was limited to a population aged 45–65 years. All individuals in the study groups were on a stable hypoglycemic, antihypertensive, lipid-lowering, and/or antiplatelet treatment regimen for at least 3 months prior to participation in the study. The exclusion criteria for the study encompassed the following: (1) other types of diabetes mellitus, (2) micro- and other macrovascular diabetic complications (retinopathy, neuropathy, nephropathy, peripheral artery disease, stroke, cerebrovascular disease) or acute diabetic complications (diabetic ketoacidosis and coma, hyperglycemic hyperosmolar state, lactic acidosis), (3) autoimmune diseases, (4) acute and chronic inflammatory diseases, (5) human immunodeficiency virus or hepatitis C virus infection, (6) liver dysfunction (aspartate aminotransferase or alanine aminotransferase levels exceeding 2 times the upper limit of the normal range), (7) renal impairment with estimated glomerular filtration rate (eGFR) < 60 mL/min/1.73 m^2^, (8) cancers, (9) previous myocardial infarction, percutaneous coronary intervention and/or coronary artery bypass grafting, (10) heart failure, (11) cardiac arrhythmias, (12) moderate to severe valvular heart disease, congenital heart disease, cardiomyopathy, (13) surgery or trauma in the past 3 months, (14) the use of drugs with the proven effect on blood counts, including glucocorticoids, in the past 6 months, (15) a daily alcohol consumption greater than or equal to 30 g for men and 20 g for women, (16) smoking.

The study protocol was approved by the Bioethics Committee of the Medical University of Lublin, Poland (No. KE-0254/198/2020) and the research was conducted in accordance with Good Clinical Practice (Declaration of Helsinki of 1975, revised in 2013). Written informed consent was obtained from all participants prior to enrollment in the study.

### Anthropometric measurements

Anthropometric parameters such as body weight and height, waist and hip circumferences were measured (to the nearest 0.1 kg and 0.1 cm) using standardized equipment and methodology. BMI was calculated as weight in kilograms divided by height in meters squared (expressed in units of kg/m^2^), and waist-to-hip ratio (WHR) as the ratio of waist circumference to hip circumference. Systolic (SBP) and diastolic (DBP) blood pressure were taken in three sets using an oscillometric method in a sitting position after a minimum of 5 min of quiet rest, and calculated as the average of the second and third measurements. A twelve lead electrocardiography was performed on all subjects and was carefully reviewed by a cardiologist for abnormalities.

### Sample collection

Whole blood (10 mL) was collected by venipuncture after a 12-hour overnight fast (between 7:30 and 8:30 a.m.) into plastic tubes (S-Monovette, Sarstedt, Nümbrecht, Germany) and processed within 30 min to obtain plasma and serum for routine laboratory analyses. Biochemical measurements, including plasma glucose, glycated hemoglobin A1c (HbA1c) and fibrinogen, serum triglycerides (TG), total cholesterol (TC), high-density lipoprotein (HDL)-C, uric acid, creatinine, high-sensitivity C-reactive protein (hs-CRP), and total homocysteine concentrations were performed by automated standard methods in a centralized laboratory using Atellica CH 930 analyzer (Siemens Healthineers, Erlangen, Germany). The concentration of low-density lipoprotein (LDL)-C was calculated according to Friedewald equation. eGFR was calculated according to Modification of Diet in Renal Disease (MDRD) formula. Complete blood counts were analyzed using an automated hematology analyzer (Yumizen H500 Analyzer, Horiba Medical, Northampton, UK).

Five millilitres of whole blood for miRNA analysis were collected in ethylenediaminetetraacetic acid (EDTA)-containing plastic tubes (S-Monovette, Sarstedt, Nümbrecht, Germany) at the same time and under the same conditions as the samples for biochemical testing. The samples were stored on ice and processed immediately after the draw using two-step centrifugation protocol: centrifugation at 3,000× *g* for 10 min at 4 °C to remove blood cells, and then centrifugation of the isolated supernatant at 16,000× *g* for 10 min at 4 °C to remove additional cellular nucleic acids attached to cell debris and to obtain platelet-poor plasma. Subsequently, the plasma was transferred to RNase/DNase-free tubes (DNA LoBind Tubes, Eppendorf, Hamburg, Germany) and stored at − 80 °C, in the absence of freeze-thaw cycles, until further analysis.

### Total RNA isolation

The total RNA, including miRNA, was extracted from 200 µl of plasma using the miRNeasy Serum/Plasma Kit (Qiagen, Valencia, CA, USA) according to the manufacturer’s instructions. The RNA concentration was determined using a NanoDrop 2000c spectrophotometer (Thermo Fisher Scientific, Waltham, MA, USA). For all analyzed samples, the A260/A280 purity ratio was between 1.8 and 2.0. In addition, the quality and integrity of RNA was assessed with an Agilent Bioanalyzer 2100 (Agilent Technologies, Santa Clara, CA, USA) and the RNA 6000 Pico Kit according to the manufacturer’s protocol. The isolated RNA was stored at − 80 °C for further analysis.

### miRNA expression profiling using microarrays

The miRNA expression profile was determined using the microarray system with the GeneChip™ miRNA 4.0 Array chip (Affymetrix, Santa Clara, CA, USA). Briefly, the total RNA (400 ng) was labeled using the FlashTag™ Biotin HSR RNA Labeling Kit (Affymetrix, Santa Clara, CA, USA) following the manufacturer’s recommendations. The labeled RNA was hybridized to gene chips in a GeneChip™ Hybridization Oven 645 (Affymetrix, Santa Clara, CA, USA) at 48 °C for 16  h. The gene chips were washed and stained using the GeneChip Hybridization Wash and Stain Kit (Affymetrix, Santa Clara, CA, USA), and then scanned with a GeneChip™ Scanner 3000 7G (Affymetrix, Santa Clara, CA, USA).

The raw intensities in a CEL format were imported into the Transcriptome Analysis Console (TAC) software version 4.0.1 (Thermo Fisher Scientific, Waltham, MA, USA), normalized using the Robust MultiArray Average (RMA) method, and processed by the Detection Above Background (DABG) algorithm to determine whether a given probeset is reasonably expressed (*p*-value < 0.05). Then, the data were log2-transformed for further analysis to determine differentially expressed miRNAs (DE-miRNAs) between patients with T2DM, T2DM-CAD, CAD and controls. miRNAs that showed fold change (FC) of >|1.5| between the study and control groups, with a *p*-value < 0.05 and an adjusted *p*-value by the Benjamini-Hochberg correction for multiple hypothesis testing (False Discovery Rate, FDR value) ≤ 0.05, were considered statistically significant and differentially expressed.

### Validation of microarray results by reverse transcription quantitative real-time polymerase chain reaction (RT-qPCR)

To confirm the expression of selected miRNAs identified by the microarray analysis, RT-qPCR was performed. In the first step, 1 µg of total RNA was reverse transcribed into first-strand cDNA using the TaqMan™ miRNA Reverse Transcription Kit (Thermo Fisher Scientific, Waltham, MA, USA) with the addition of an RNase inhibitor (Thermo Fisher Scientific, Waltham, MA, USA). The reaction mixture was then incubated at 16 °C for 30 min, at 42 °C for 30 min and at 85 °C for 5 min in a Veriti™ Dx 96-well Thermal Cycler (Applied Biosystems, Thermo Fisher Scientific, Foster City, CA, USA), and then held at 4 °C. cDNA samples were stored at − 20 °C until analysis.

In the next step, quantitative Real-Time PCR (qPCR) was carried out using the TaqMan™ Universal PCR Master Mix (Applied Biosystems, Thermo Fisher Scientific, Waltham, MA, USA) in a StepOnePlus™ Real-Time PCR System (Applied Biosystems, Thermo Fisher Scientific, Waltham, MA, USA) according to the manufacturer’s protocol, where the premix of cDNA was used as a template for relative quantification of miRNAs. The sequences of five specific miRNA primers (hsa-miR-3613-3p, hsa-miR-4505, hsa-miR-4668-5p, hsa-miR-4743-5p, hsa-miR-4750-3p) (TaqMan™ MicroRNA Assays; Applied Biosystems, Thermo Fisher Scientific, Waltham, MA, USA) used for RT-qPCR are shown in Table 1.

**Table 1:** List of primer sequences for RT-qPCR

Assay name	Assay ID	miRBase ID^1^	miRBase Accession Number^1^	Primer sequence (5’ to 3’)
hsa-miR-3613-3p	463183_mat	hsa-miR-3613-3p	MIMAT0017991	ACAAAAAAAAAAGCCCAACCCUUC
hsa-miR-4505	464775_mat	hsa-miR-4505	MIMAT0019041	AGGCUGGGCUGGGACGGA
hsa-miR-4668-5p	465086_mat	hsa-miR-4668-5p	MIMAT0019745	AGGGAAAAAAAAAAGGAUUUGUC
hsa-miR-4743-5p	462505_mat	hsa-miR-4743-5p	MIMAT0019874	UGGCCGGAUGGGACAGGAGGCAU
hsa-miR-4750-3p	475980_mat	hsa-miR-4750-3p	MIMAT0022979	CCUGACCCACCCCCUCCCGCAG
U6 snRNA	001973	715680	NR_004394^2^	GTGCTCGCTTCGGCAGCACATATACTAAAATTGGAACGATACAGAGAAGATTAGCATGGCCCCTGCGCAAGGATGACACGCAAATTCGTGAAGCGTTCCATATTTT
RNU6B	001093	568915	NR_002752^2^	CGCAAGGATGACACGCAAATTCGTGAAGCGTTCCATATTTTT
RNU48	001006	568908	NR_002745^2^	GATGACCCCAGGTAACTCTGAGTGTGTCGCTGATGCCATCACCGCAGCGCTCTGACC

^1^According to miRBase 22 (https://www.mirbase.org/)

^2^NCBI Accession Number

The reactions were incubated in a 96-well MicroAmp Fast Optical 0.1 mL reaction plate (Applied Biosystems, Thermo Fisher Scientific, Waltham, MA, USA) at 50 °C for 2 min and at 95 °C for 10 min, followed by 40 cycles of PCR amplification at 95 °C for 15 s, and elongation at 60 °C for 1 min. All the PCR reactions were run in triplicate.

The relative expression (RQ) of each individual miRNA was calculated using the 2^−ΔΔCt^ method, and logarithmically transformed (log_10_RQ) to reduce skewness [22]. To normalize the expression levels of candidate miRNAs, the cycle threshold (Ct) value was determined for each sample against the reference gene, small nuclear RNA (snRNA) U6. In the study, RNU6B (RNU6-6P) and RNU48 (SNORD48) were also considered as endogenous controls due to their relatively constant and highly abundant expression across human tissues and cell line types, and a wide use in various fields, including diabetes and coronary artery disease research, but U6 showed the most stable expression in the tested material, and therefore proved to be the best control. The data were analyzed with Expression Suite Software version 1.0.3. (Applied Biosystems, Thermo Fisher Scientific, Waltham, MA, USA) with the automatic Ct setting for assigning baseline and threshold for Ct determination.

### Functional enrichment analysis of differentially expressed miRNAs

The web-based DNA Intelligent Analysis (DIANA)-miRPath v3.0 tool (https://dianalab.e-ce.uth.gr/html/mirpathv3/index.php?r=mirpath, accessed on 31 May 2023) was used to perform functional enrichment analysis for each of the DE-miRNAs in Kyoto Encyclopedia of Genes and Genomes (KEGG) pathways and Gene Ontology (GO) terms [23]. The target genes of DE-miRNAs were obtained by combining available *in silico* predicted targets from the DIANA-microT-CDS v5.0 and/or TargetScan v6.2 databases and high quality, manually curated and experimentally validated targets from the DIANA-TarBase v7.0 database [23]. The *p*-values were calculated with Fisher’s exact test, and adjusted using FDR correction for multiple comparisons. The cut-off criterion was a *p*-value limited to 0.05. Visualization of the miRNA regulatory network with appropriate KEGG and/or GO annotations was carried out using Cytoscape v3.10.0 software (https://cytoscape.org/, accessed on 15 June 2023) [24].

The analysis of the interactions of selected miRNAs with genes was conducted in the R environment v4.3.1 (https://www.r-project.org, accessed on 20 June 2023) using the multiMiR 1.23.0 package (https://bioconductor.org/packages/release/bioc/html/multiMiR.html, accessed on 20 June 2023) [25]. This package retrieved miRNA-target interactions from 14 external databases, which include predicted (DIANA-microT-CDS, ElMMo, MicroCosm, miRanda, miRDB, PicTar, PITA, TargetScan) and experimentally validated (miRecords, miRTarBase, TarBase) interactions and miRNA-drug/disease associations (miR2Disease, Pharmaco-miR, PhenomiR) [25]. The intersection of the results of these databases was considered as the final prediction of DE-miRNA target genes.

Subsequently, an enrichment analysis was performed using the miRNet 2.0 tool (https://www.mirnet.ca/miRNet/home.xhtml, accessed on 25 June 2023), where a list of selected miRNAs and a list of genes with which they interact were introduced [26]. KEGG and Reactome pathways and GO categories were searched to understand the biological relevance of DE-miRNA target genes in T2DM-CAD. The terms with an adjusted *p*-value < 0.05 was regarded as statistically significant. The plot visualizing the enrichment analysis was generated using the ggplot2 3.3.0 package in the R environment.

### Protein-protein interaction networks and sub-networks construction

To establish the regulatory role of validated DE-miRNA target genes in T2DM-CAD, the Search Tool for the Retrieval of Interacting Genes (STRING) App v2.0.1 (https://apps.cytoscape.org/apps/stringapp, accessed on 25 July 2023) in Cytoscape v3.10.0 software was used to construct the protein-protein interaction (PPI) networks [27]. Interactions between proteins encoded by co-expressed genes, including direct (physical) and indirect (functional) associations, were obtained from different sources, and only interactions with a combined confidence score greater than 0.4 were retained.

Then, the Molecular Complex Detection (MCODE) v2.0.3 (https://apps.cytoscape.org/apps/mcode, accessed on 25 July 2023) plugin of Cytoscape v3.10.0 tool was used to gain insights into possible closely connected gene modules (clusters, sub-networks) within the PPI networks and perform an enrichment analysis [28]. The networks were clustered based on inflation parameters according to the Markov Clustering (MCL) algorithm, and the 6 top-scored clusters separately for up- and down-regulated miRNAs were analyzed. The selection criteria were as follows: cluster finding = hair-cut, node score cut-off = 0.2, degree cut-off = 2, k-core filter of 2 and maximum depth = 100. In the networks, each node represents a protein produced by a single, protein-coding gene locus, and edges indicate both functional and physical associations of the proteins.

### Hub gene identification

To identify the key genes (hub genes) in the PPI networks, the CytoHubba plugin (https://apps.cytoscape.org/apps/cytohubba, accessed on 25 July 2023) of Cytoscape v3.10.0 software was applied [29]. The top 10 ranked hub genes were extracted using the BottleNeck algorithm according to their betweenness centrality. Next, an enrichment analysis was conducted to forecast the function of identified hub genes of the T2DM-CAD gene regulatory networks.

### Statistical analysis

Sample size calculation was based on similar previous experiments and pilot data on microarray miRNA expression profiling in patients with T2DM and CAD [30–33]. We used the MD Anderson sample size calculator (http://bioinformatics.mdanderson.org/MicroarraySampleSize) to estimate the minimum number of samples per experimental groups (T2DM-CAD and T2DM) to detect 1.5 fold differences in miRNA expression levels between groups at the true positive detection power (1-β) of 80% [34–36]. The sample size of 12 patients for each of the 2 groups was calculated to provide 80% power with a significance level (α) set at 5% and a standard deviation (SD) of 0.5.

Categorical variables are expressed as numbers with percentages (%). Continuous data are presented as mean ± SD or median with interquartile range (IQR), depending on the normality of distribution assessed by the Shapiro–Wilk test. Parametric tests were used for variables with normal distributions. Student’s *t*-test was used to compare differences between two groups, whereas one-way analysis of variance (ANOVA) test was used for more than 2 groups with Tukey’s post hoc test for unequal *n*. Data conforming to non‑normal distribution were analyzed using the Mann-Whitney *U* test (for comparisons of two groups) and the Kruskal-Wallis one-way analysis of variance by ranks followed by Dunn’s test (for comparisons of more than two groups). Chi-Squared test was performed to determine sex distribution between groups.

To estimate the diagnostic significance of particular DE-miRNAs in detecting CAD in T2DM patients, receiver operating characteristic (ROC) analysis was performed, and the area under the curve (AUC) was calculated. Multivariate logistic regression analysis using the generalized linear model (GLM) function included in R was used to train miRNA diagnostic models. The expression value of identified miRNAs was considered as predictive variables, and the sample type (T2DM-CAD or T2DM) was considered as a binary response variable. To assess the diagnostic accuracy of each multi-miRNA panel, ROC curves and AUC were calculated by the pROC package. The cut-off points for ROC curves were calculated by the Youden index.

All *p*-values are two-tailed, and *p*-values < 0.05 were considered as statistically significant. The statistical analysis was performed with STATISTICA 13.3.1. software for Windows (TIBCO Software Inc., Palo Alto, CA, USA), R Studio version 4.3.1 (R Studio, Boston, MA, USA), and GraphPad Prism 10.2.0 software for Windows (GraphPad Software, San Diego, CA, USA).

## Results

### Baseline characteristics of study and control groups

A total of 132 patients meeting eligibility criteria were prospectively recruited for the miRNA profiling study (*n* = 38) and the validation cohort (*n* = 94). All participants in both cohorts were divided into four groups (T2DM-CAD, T2DM, CAD, controls) matched for age, sex, and BMI. As expected, patients with T2DM had significantly higher HbA1c levels and fasting plasma glucose concentrations compared to non-T2DM individuals (*p* < 0.001). None of the study groups differed in a series of clinical and biochemical parameters (SBP, DPB, TC, TG, LDL-C, hs-CRP, platelets, fibrinogen, uric acid, and renal function), indicating that confounding factors related to vascular complications were excluded. Only HDL-C, homocysteine concentrations, and white blood cell counts differed significantly between all four groups in both the discovery and validation cohorts.

All T2DM patients received combination therapy comprising a biguanide derivative (metformin) and sodium-glucose co-transporter type 2 inhibitors (SGLT-2is) or glucagon-like peptide-1 receptor agonists (GLP-1RAs). Participants diagnosed with CAD, either with or without T2DM, were treated with low-dose aspirin and did not receive any other antiplatelet medications. Additionally, approximately 90% of individuals with T2DM and all patients with CAD in the discovery and validation cohorts received statins, resulting in normal lipid parameters or, in some cases, only mild dyslipidemia. The frequency of hypertension was higher among patients with T2DM-CAD (100%) and CAD (100%) compared to those with T2DM (91.67% vs. 96.67%) in the discovery and validation phase of the study (*p* = 0.423 and *p* = 0.460). No significant intergroup differences were observed with respect to the antihypertensive treatment regimen used.

The demographic and clinical characteristics of patients enrolled in the discovery and validation cohorts are summarized in Table 2.

**Table 2:** Baseline characteristics of participants in the discovery and validation cohorts

	Discovery cohort					Validation cohort				
Variables	T2DM-CAD (*n* = 12)	T2DM (*n* = 12)	CAD (*n* = 8)	Controls (*n* = 6)	*p*-Value	T2DM-CAD (*n* = 30)	T2DM (*n* = 30)	CAD (*n* = 16)	Controls (*n* = 18)	*p*-Value
Age [years]	58.67 ± 3.82	56.92 ± 2.19	58.63 ± 2.50	55.33 ± 3.27	0.114 ^a^	58.27 ± 4.31	57.03 ± 4.46	58.19 ± 2.88	55.61 ± 4.13	0.146 ^a^
Female, *n* (%)	6 (50)	6 (50)	4 (50)	3 (50)	1.000 ^b^	18 (60.00)	20 (66.67)	7 (43.75)	10 (55.56)	0.503 ^b^
Male, *n* (%)	6 (50)	6 (50)	4 (50)	3 (50)		12 (40.00)	10 (33.33)	9 (56.25)	8 (44.44)	
BMI [kg/m^2^]	28.40 ± 3.77	30.43 ± 1.46	27.84 ± 1.58	28.10 ± 3.29	0.137 ^a^	29.58 ± 4.08	31.09 ± 2.94	28.56 ± 3.00	29.67 ± 4.22	0.130 ^a^
WHR	0.99 ± 0.11	1.00 ± 0.07	0.95 ± 0.04	0.95 ± 0.14	0.603 ^a^	0.98 ± 0.09	0.97 ± 0.09	0.95 ± 0.08	0.93 ± 0.10	0.282 ^a^
Duration of T2DM [years]	13.00 ± 5.27	12.58 ± 6.78	–	–	0.868 ^c^	12.50 ± 5.40	11.57 ± 5.20	–	–	0.498 ^c^
HbA1c [%]	7.68 ± 0.81 ^1,2***^	7.08 ± 0.73 ^1,2***^	5.29 ± 0.30	5.03 ± 0.10	< 0.001 ^a***^	7.47 ± 0.78 ^1,2***^	7.42 ± 1.02 ^1,2***^	5.31 ± 0.25	5.24 ± 0.25	< 0.001 ^a***^
FPG [mg/dL]	135.25 ± 24.39 ^1,2**^	132.50 ± 29.23 ^1*,2**^	93.25 ± 3.77	90.83 ± 3.60	< 0.001 ^a***^	138.37 ± 27.58 ^1,2***^	137.43 ± 31.70 ^1,2***^	91.44 ± 5.07	89.72 ± 5.11	< 0.001 ^a***^
TC [mg/dL]	154.83 ± 44.94	155.75 ± 34.69	142.63 ± 38.64	180.33 ± 18.46	0.329 ^a^	149.47 ± 38.06	161.70 ± 34.68	156.00 ± 33.06	175.17 ± 14.29	0.072 ^a^
TG [mg/dL]	129.83 ± 47.97	124.67 ± 29.05	130.38 ± 46.29	107.00 ± 30.94	0.677 ^a^	137.17 ± 47.91	140.77 ± 45.85	123.00 ± 43.92	113.11 ± 38.28	0.156 ^a^
LDL-C [mg/dL]	81.78 ± 36.26	89.73 ± 29.63	77.18 ± 30.88	100.93 ± 17.06	0.491 ^a^	78.40 ± 31.27	91.98 ± 30.79	84.90 ± 27.00	97.60 ± 13.95	0.099 ^a^
HDL-C [mg/dL]	47.08 ± 10.28	41.08 ± 10.08	39.38 ± 6.05 ^1*^	58.00 ± 17.37	0.012 ^a*^	43.63 ± 9.67 ^1*^	41.57 ± 11.10 ^1**^	46.50 ± 13.28	54.94 ± 14.16	0.002 ^a**^
WBC [10^9^/L]	7.00 ± 1.44 ^1*^	6.26 ± 0.75	6.73 ± 1.33	5.01 ± 0.72	0.011 ^a^	6.72 ± 1.47	6.64 ± 1.20	6.91 ± 1.48	5.68 ± 1.27	0.032 ^a*^
hs-CRP [mg/L]	1.27 (0.55–1.78)	1.18 (0.50–3.96)	1.54 (0.62–2.18)	0.64 (0.50–1.04)	0.467 ^d^	1.37 (0.89–2.22)	1.29 (0.50–6.24)	1.31 (0.50–2.04)	0.75 (0.50–1.04)	0.285 ^d^
PLT [10^9^/L]	221.92 ± 44.06	239.33 ± 54.32	231.63 ± 57.43	203.67 ± 20.29	0.503 ^a^	224.47 ± 42.58	236.33 ± 60.42	250.06 ± 59.94	244.00 ± 52.03	0.414 ^a^
Fibrinogen [mg/dL]	3.05 (2.65–3.45)	3.40 (3.00–3.65)	3.75 (2.95–4.90)	2.60 (2.40–2.70)	0.054 ^d^	3.10 (2.70–3.60)	3.10 (2.90–3.70)	3.20 (2.70–4.00)	3.05 (2.60–3.40)	0.530 ^d^
Homocysteine [µmol/L]	21.15 ± 4.17	20.48 ± 4.65	17.49 ± 3.75	15.18 ± 3.35	0.023 ^a^*	20.56 ± 4.83 ^1,3**^	16.53 ± 5.42	18.07 ± 3.25	15.32 ± 3.71	0.001 ^a***^
Creatinine [mg/dL]	0.74 ± 0.12	0.78 ± 0.16	0.86 ± 0.10	0.78 ± 0.08	0.220 ^a^	0.80 ± 0.12	0.73 ± 0.14	0.83 ± 0.09	0.80 ± 0.14	0.054 ^a^
eGFR [mL/min/1.73m2]	90.00 (84.85–90.00)	90.00 (82.90–90.00)	78.63 (71.50–89.85)	90.00 (85.70–90.00)	0.131 ^d^	86.70 (75.00–90.00)	90.00 (78.10–90.00)	88.17 (78.63–90.00)	90.00 (78.17–90.00)	0.550 ^d^
Uric acid [mg/dL]	5.23 ± 1.17	5.37 ± 1.12	5.95 ± 1.51	5.02 ± 1.36	0.524 ^a^	5.28 ± 1.41	5.80 ± 1.36	5.51 ± 1.39	5.06 ± 1.04	0.252 ^a^
SBP [mmHg]	131.50 ± 6.45	133.33 ± 8.98	134.38 ± 5.55	128.17 ± 6.34	0.401 ^a^	129.30 ± 7.70	129.83 ± 6.30	129.69 ± 9.71	126.83 ± 6.06	0.552 ^a^
DBP [mmHg]	75.33 ± 6.05	75.92 ± 7.19	78.00 ± 4.54	79.50 ± 3.62	0.468 ^a^	76.73 ± 7.26	79.77 ± 5.45	77.19 ± 6.24	81.22 ± 5.16	0.055 ^a^
Metformin, *n* (%)	12 (100.00)	12 (100.00)	–	–	1.000 ^b^	30 (100.00)	30 (100.00)	–	–	1.000 ^b^
SGLT-2is, *n* (%)	9 (75.00)	10 (83.33)	–	–	0.615 ^b^	24 (80.00)	23 (76.67)	–	–	0.754 ^b^
GLP-1RAs, *n* (%)	3 (25.00)	2 (16.77)	–	–	0.615 ^b^	6 (20.00)	7 (23.33)	–	–	0.754 ^b^
Aspirin, *n* (%)	12 (100.00)	–	8 (100.00)	–	1.000 ^b^	30 (100.00)	–	16 (100.00)	–	1.000 ^b^
ACEIs, *n* (%)	6 (50.00)	6 (50.00)	5 (62.50)	–	0.828 ^b^	15 (50.00)	15 (50.00)	8 (50.00)	–	1.000 ^b^
ARBs, *n* (%)	5 (41.67)	4 (33.33)	3 (37.50)	–	0.915 ^b^	12 (40.00)	12 (40.00)	7 (43.75)	–	0.964 ^b^
CCBs, *n* (%)	8 (66.67)	6 (50.00)	6 (75.00)	–	0.491 ^b^	16 (53.33)	17 (56.67)	9 (56.25)	–	0.963 ^b^
β-blockers, *n* (%)	12 (100.00)	9 (75.00)	8 (100.00)	–	0.063 ^b^	24 (80.00)	17 (56.67)	12 (75.00)	–	0.127 ^b^
Statins, *n* (%)	12 (100.00)	11 (91.67)	8 (100.00)	–	0.423 ^b^	28 (93.33)	27 (90.00)	15 (93.75)	–	0.859 ^b^

Data are presented as number (%) and mean ± SD or median (IQR) based on the data distribution

T2DM-CAD, type 2 diabetes mellitus with coronary artery disease; T2DM, type 2 diabetes mellitus; CAD, coronary artery disease; BMI, body mass index; WHR, waist-to-hip ratio; HbA1c, glycated hemoglobin A1c; FPG, fasting plasma glucose; TC, total cholesterol; TG, triglycerides; LDL-C, low-density lipoprotein cholesterol; HDL-C, high-density lipoprotein cholesterol; WBC, white blood cell; hs-CRP, high-sensitivity C-reactive protein; PLT, platelet; eGFR, estimated glomerular filtration rate; SBP, systolic blood pressure; DBP, diastolic blood pressure; SGLT-2is, sodium-glucose co-transporter type 2 inhibitors; GLP-1RAs, glucagon-like peptide-1 receptor agonists; ACEIs, angiotensin-converting enzyme inhibitors; ARBs, angiotensin II receptor blockers; CCBs, calcium channel blockers

^1^ Significantly different from controls; ^2^ Significantly different from CAD; ^3^ Significantly different from T2DM. *p*-value < 0.05 was statistically significant. *p < 0.05; **p < 0.01; ***p < 0.001. ^a^ One-way ANOVA with Tukey’s post hoc test for unequal *n*; ^b^ Chi-Squared test; ^c^ Student’s *t*-test; ^d^ Kruskal-Wallis one-way ANOVA by ranks test followed by Dunn’s test for more than two groups

### Screening for differentially expressed circulating miRNAs

To determine the DE-miRNAs in the plasma of T2DM patients with CAD (*n* = 12) compared to individuals with T2DM (*n* = 12), patients with CAD (*n* = 8), and the control group (*n* = 6), we performed the expression analysis of 2,578 unique miRNAs using a microarray system. The established threshold criteria (*p*-value < 0.05, FDR ≤ 0.05 and FC > 1.5 or < -1.5) yielded a list of 18 DE-miRNAs, of which 3 were up-regulated and 15 were down-regulated between the T2DM-CAD and T2DM groups (**Additional file 1: Table ****S1**). A total of 35 DE-miRNAs were found between T2DM-CAD patients and controls (**Additional file 1: Table S2**). Additionally, 5 DE-miRNAs were identified between T2DM individuals and healthy controls (**Additional file: Table S3**), whereas 4 DE-miRNAs were detected between the T2DM-CAD and CAD groups (**Additional file 1: Table S4**). Finally, the comparisons of those sets of DE-miRNAs (**Additional file 1: Figure ****S1**) allowed to extract 12 DE-miRNAs typical only for T2DM patients with CAD (Table 3).

**Table 3:** Significantly dysregulated miRNAs between the T2DM-CAD and T2DM groups in the discovery cohort

miRNA	T2DM-CAD		T2DM		FC	*p*-Value	FDR
	*n* = 12		*n* = 12				
hsa-miR-4505	8.15 ± 2.74	7.13 (6.15–10.90)	2.55 ± 2.74	1.22 (0.96–3.83)	118.00	< 0.001 ^1^	< 0.001
hsa-miR-4743-5p	4.11 ± 2.45	3.68 (1.63–6.61)	1.73 ± 1.80	1.37 (0.74–1.82)	6.85	0.014 ^1^	0.02
hsa-miR-6846-5p	2.99 ± 1.66	2.00 (1.66–4.92)	1.62 ± 1.36	1.14 (0.90–1.76)	1.95	0.008 ^1^	0.01
hsa-miR-7850-5p	0.77 ± 0.21	0.76 (0.62–0.88)	1.45 ± 0.36	1.50 (1.15–1.74)	− 1.66	< 0.001 ^2^	< 0.001
hsa-miR-4717-3p	1.05 ± 0.45	1.07 (0.74–1.43)	1.92 ± 0.49	1.92 (1.54–2.31)	− 1.79	< 0.001 ^2^	< 0.001
hsa-miR-320e	6.05 ± 0.58	6.02 (5.73–6.47)	6.57 ± 0.74	6.88 (6.11–7.02)	− 1.83	0.039 ^1^	0.04
hsa-miR-4750-3p	1.35 ± 0.37	1.18 (1.08–1.62)	2.28 ± 0.78	2.19 (1.66–2.68)	− 2.02	0.001 ^1^	< 0.01
hsa-miR-6750-5p	2.49 ± 0.64	2.52 (1.98–2.96)	3.50 ± 0.71	3.58 (2.99–4.03)	− 2.06	0.001 ^2^	< 0.01
hsa-miR-6511b-5p	2.37 ± 0.90	2.44 (1.59–3.11)	3.87 ± 1.02	3.58 (2.99–4.77)	− 2.50	0.001 ^2^	< 0.01
hsa-miR-4706	2.85 ± 0.74	2.73 (2.32–3.29)	4.06 ± 0.99	4.23 (3.35–4.91)	− 2.67	0.003 ^2^	0.01
hsa-miR-4668-5p	2.91 ± 2.27	1.68 (1.13–4.58)	6.16 ± 3.05	7.17 (4.29–8.28)	− 66.16	0.017 ^1^	0.02
hsa-miR-3613-3p	2.71 ± 2.46	1.41 (0.84–4.28)	6.66 ± 3.23	7.79 (4.13–9.03)	− 103.27	0.002 ^1^	0.01

Values are presented as mean ± SD and median (interquartile range, IQR)

T2DM-CAD, type 2 diabetes mellitus with coronary artery disease; T2DM, type 2 diabetes mellitus; FC, fold change; FDR, false discovery rate

^1^ Mann–Whitney *U* test; ^2^ Student’s *t*-test. *p*-value < 0.05 and FDR ≤ 0.05 were statistically significant. FC > 1.5 or < -1.5

Among them, 3 miRNAs (hsa-miR-4505, hsa-miR-4743-5p, hsa-miR-6846-5p) were up-regulated and 9 miRNAs (hsa-miR-3613-3p, hsa-miR-4668-5p, hsa-miR-4706, hsa-miR-6511b-5p, hsa-miR-6750-5p, hsa-miR-4750-3p, hsa-miR-320e, hsa-miR-4717-3p, hsa-miR-7850-5p) were down-regulated in T2DM-CAD patients compared to T2DM individuals (**Additional file 1: Figure S2**).

Moreover, we evaluated the diagnostic value of DE-miRNAs as candidate biomarkers for T2DM-CAD. All identified DE-miRNAs showed good ability to effectively differentiate CAD in T2DM patients with AUC values greater than 0.700 (all *p* < 0.05). The highest discriminatory power was obtained for hsa-miR-7850-5p (AUC = 0.969), hsa-miR-4505 (AUC = 0.938), hsa-miR-4717-3p (AUC = 0.913) and hsa-miR-4750-3p (AUC = 0.889) (**Additional file 1: Figure S2**).

### Functional annotation and pathway enrichment analysis

**Fig. 2:**
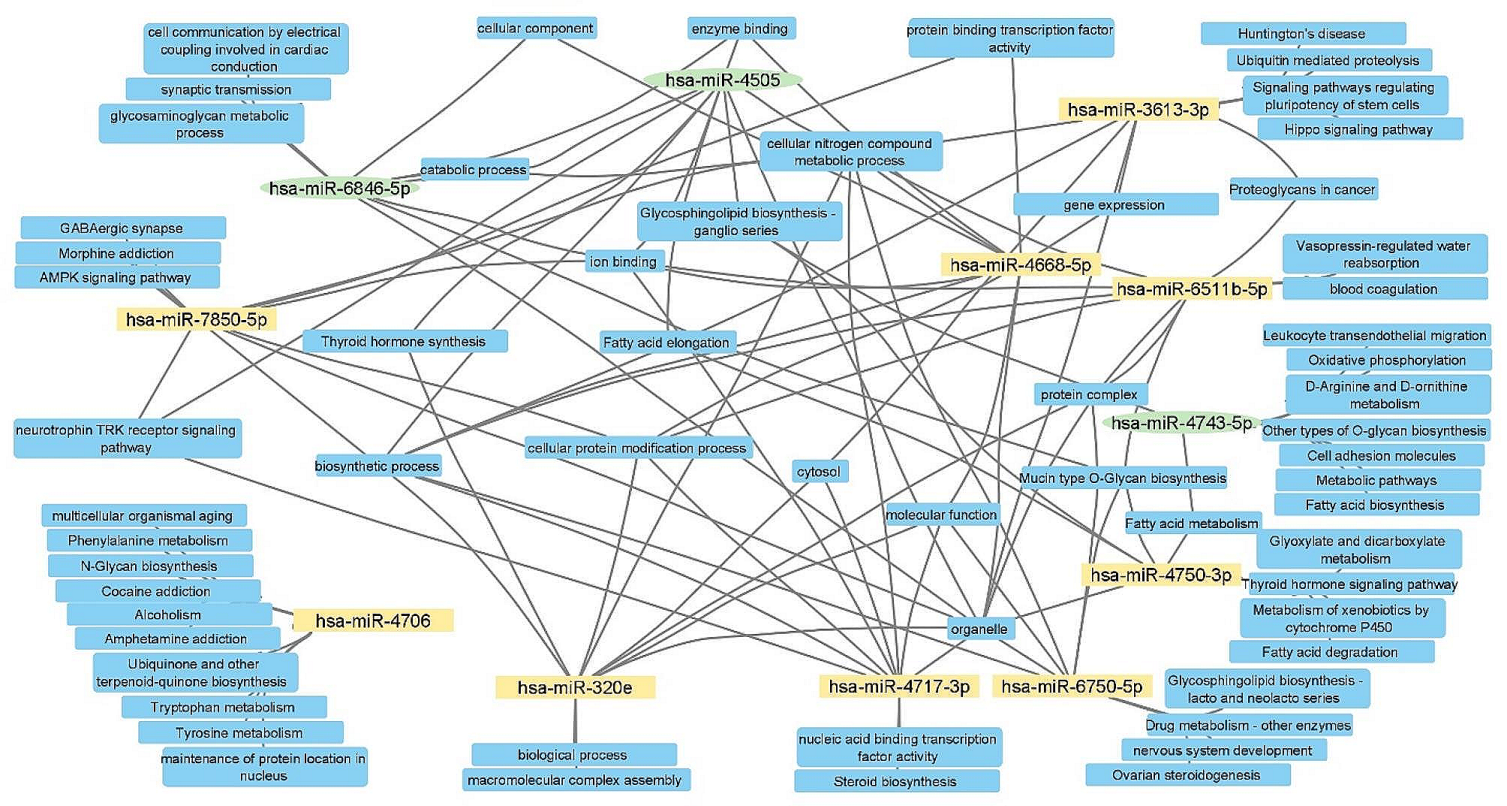
Functional network of DE-miRNAs in T2DM-CAD along with their top 10 pathways and biological features. Down-regulated miRNAs are marked in yellow, while up-regulated miRNAs are marked in green. Analysis was performed using the DIANA-miRPath v3.0 tool. DE-miRNA, differentially expressed miRNA; T2DM-CAD, type 2 diabetes mellitus with coronary artery disease

As a particular miRNA may act on several targets, the DIANA-miRPath v3.0 tool was used to identify the top 10 most significant KEGG pathways and GO annotations corresponding to gene targets for each of the 12 DE-miRNAs between patients with T2DM-CAD and T2DM. Subsequently, possible relationships between the selected up- and down-regulated miRNAs were visualized using Cytoscape v3.10.0 software. The interaction network functionally linked the 11 miRNAs together, leaving only one down-regulated hsa-miR-4706 without functional linkage (Fig. 2).

The functional network revealed that ‘ion binding’, ‘catabolic process’, ‘enzyme binding’, ‘organelle’, ‘cellular nitrogen compound metabolic process’, ‘glycosphingolipid biosynthesis– ganglio series’ were the terms reaching the most connections between up-regulated miRNAs, whereas down-regulated miRNAs were significantly enriched in terms of ‘organelle’, ‘cellular nitrogen compound metabolic process’, ‘biosynthetic process’, ‘cellular protein modification process’, ‘ion binding’, and ‘molecular function’. Among pathways strictly related to diabetic atherosclerosis, we observed enrichment of neurotrophin tropomyosin kinase (TRK) receptor signaling pathway (hsa-miR-4505, hsa-miR-4717-3p, hsa-miR-7850-5p) and processes associated with fatty acid metabolism (hsa-miR-4505, hsa-miR-4743-5p, hsa-miR-4750-3p).

**Fig. 3:**
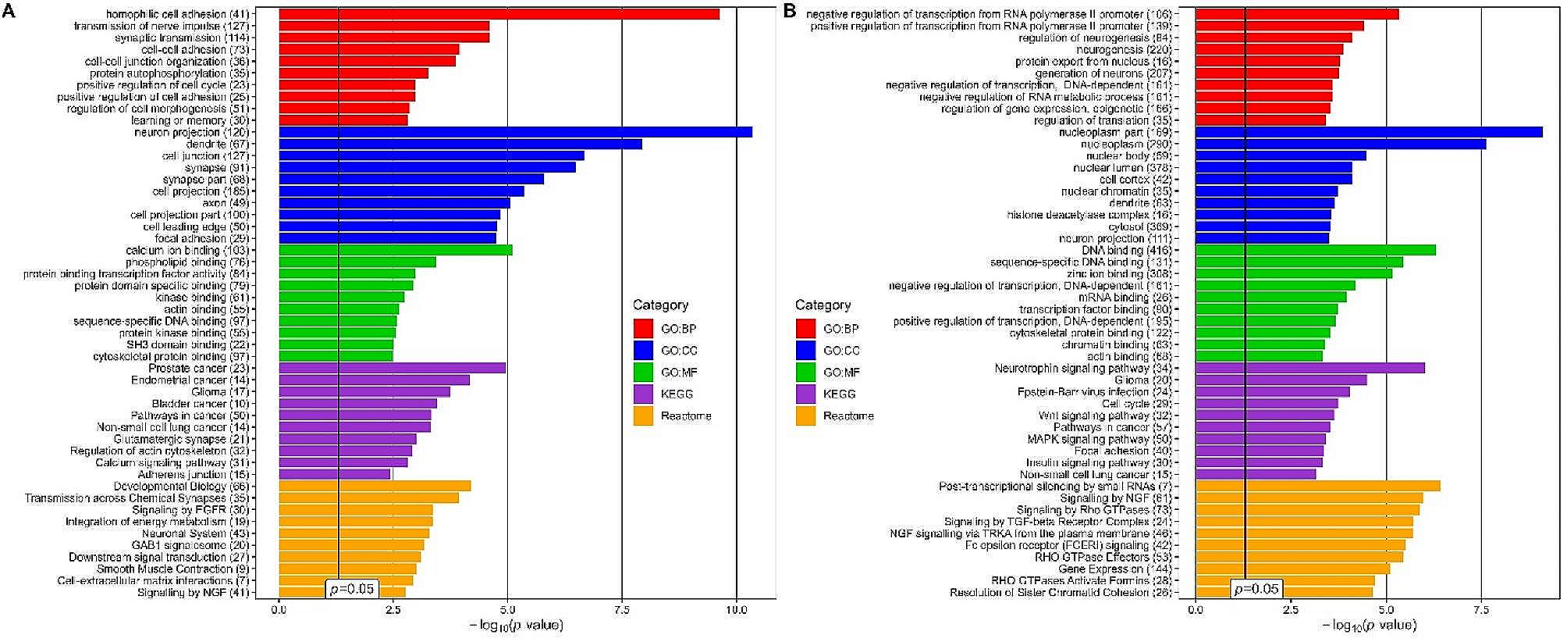
Functional enrichment analysis for genes targeted by DE-miRNAs between T2DM-CAD and T2DM. The top 10 terms of the three Gene Ontology (GO) subcategories: Biological Process (GO: BP), Cellular Component (GO: CC), Molecular Function (GO: MF) and Kyoto Encyclopedia of Genes and Genomes (KEGG) and Reactome pathways are presented separately for (**A**) up-regulated miRNAs and (**B**) down-regulated miRNAs. The terms are sorted for each category in descending order according to the adjusted *p*-values. *p*-value– EASE score for enrichment adjusted by the Benjamini-Hochberg correction for multiple hypothesis testing. Adjusted *p*-value < 0.05 was statistically significant. The number in brackets following the term names indicates the number of enriched target genes. The plot was generated using the ggplot2 3.3.0 package in the R environment. DE-miRNA, differentially expressed miRNA; T2DM-CAD, type 2 diabetes mellitus with coronary artery disease; T2DM, type 2 diabetes mellitus

To further recognize the specific biological functions of DE-miRNA-regulated target genes, functional enrichment analysis was performed separately for up- and down-regulated miRNA sets using the miRNet 2.0 tool. The top 10 KEGG and Reactome pathways and GO terms categorized as the biological process (BP), cellular component (CC), molecular function (MF), potentially relevant to the development of CAD in T2DM, are presented in Fig. 3. The gene targets of the up-regulated miRNAs were significantly overrepresented in terms related to pathways in cancers, signal transduction, mainly calcium, epidermal growth factor receptor (EGFR) and nerve growth factor (NGF) signaling pathways, metabolism, calcium ion and protein binding, cell-cell communication, cell adhesion, and cell junction organization (Fig. 3A). In the group of down-regulated miRNAs, the most prominent pathways were neurotrophin signaling pathway along with the signaling pathways of Wnt and mitogen-activated protein kinases (MAPK) and others associated with signal transduction via NGF, Rho GTPases and transforming growth factor-β (TGF-β) receptor complex. What is more, they have been found to regulate processes related to transcription and translation, the endocrine system, especially the insulin signaling pathway, cellular community, including focal adhesion, and zinc ion binding (Fig. 3B).

### Protein-protein interaction network construction

The PPI networks of the validated target genes of 12 DE-miRNAs were constructed separately for up- and down-regulated miRNAs between the T2DM-CAD and T2DM groups (**Additional file 1: Table S5**). Using the STRING database in Cytoscape v3.10.0 software, we identified 319 nodes and 484 edges, with a PPI enrichment *p*-value of 5.3 × 10^−4^ for up-regulated miRNAs, while a total of 1,710 nodes and 12,287 edges, with a PPI enrichment *p*-value < 1.0 × 10^−16^, were recognized for down-regulated miRNAs.

**Fig. 4:**
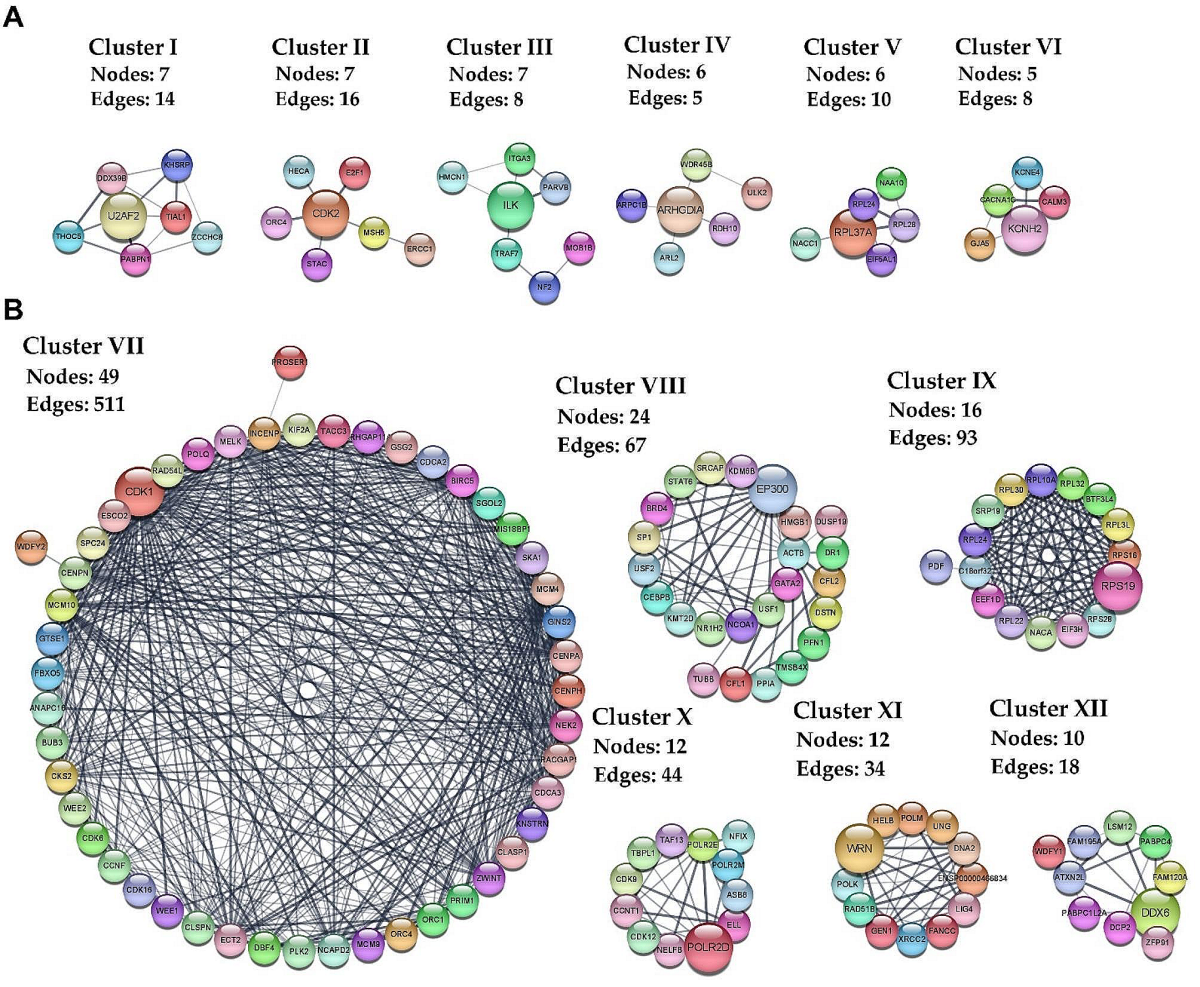
Analysis of the PPI interaction network for DE-miRNA target genes in T2DM-CAD. The 6 top-scored clusters selected from the PPI network for both (**A**) up-regulated and (**B**) down-regulated miRNAs are presented along with the number of nodes, edges, and key gene (highlighted by enlarging) in each cluster. Nodes having different colors indicate different proteins encoded by the co-expressed genes. Gray lines connect proteins within the PPI sub-networks with darker colors and thicker lines indicating higher core PPI values. The analysis was performed using the STRING database with the MCODE plugin of Cytoscape v3.10.0. PPI, protein-protein interaction; DE-miRNA, differentially expressed miRNA; T2DM-CAD, type 2 diabetes mellitus with coronary artery disease

Next, a clustering analysis of all nodes and edges was performed using the MCODE algorithm in Cytoscape v3.10.0, and the 6 highest ranked clusters for both up- (clusters I–VI) and down-regulated (clusters VII–XII) miRNAs are shown in Fig. 4.

In addition, a functional enrichment analysis was conducted to gain insights into the biomolecular significance of each cluster’s genes (**Additional File 1: Table S6** and **Table S7**). Cluster I was associated with RNA metabolism, while clusters V, IX and XI were found to be functionally linked to multiple steps in transcription and translation processes. Clusters II and VII were predominantly involved in the cell cycle. As expected, DE-miRNA target genes, grouped into sub-networks III–IV, VI, VIII–XII, were strictly enriched in various pathways underlying the pathogenesis of diabetic atherosclerosis. Cluster III was connected with the Hippo-signaling pathway, cell-extracellular matrix-interactions and, together with cluster IX, was associated with focal adhesion. Cluster IV focused on the function of the phagophore assembly site membrane, which is the main structure involved in the induction of autophagy. Cluster VI members dealt with cation, mainly calcium channel complex and the regulation of cation transmembrane transport, whereas cluster X was functionally related to ion binding. Interestingly, cluster VIII was linked to the regulation of metabolic processes, vascular endothelial cell proliferation and migration, response to oxygen-containing compounds, leukocyte activation, platelet degranulation, and lipid homeostasis. The results of the functional enrichment analysis revealed that cluster XI was involved in signaling through the TGF-beta receptor complex by regulating the transcriptional activity of the SMAD2/SMAD3:SMAD4 heterotrimer, while cluster XII was associated with the cytoplasmic stress granule, which are dynamic cytoplasmic aggregates formed in response to cellular stress.

### Hub gene identification

**Fig. 5:**
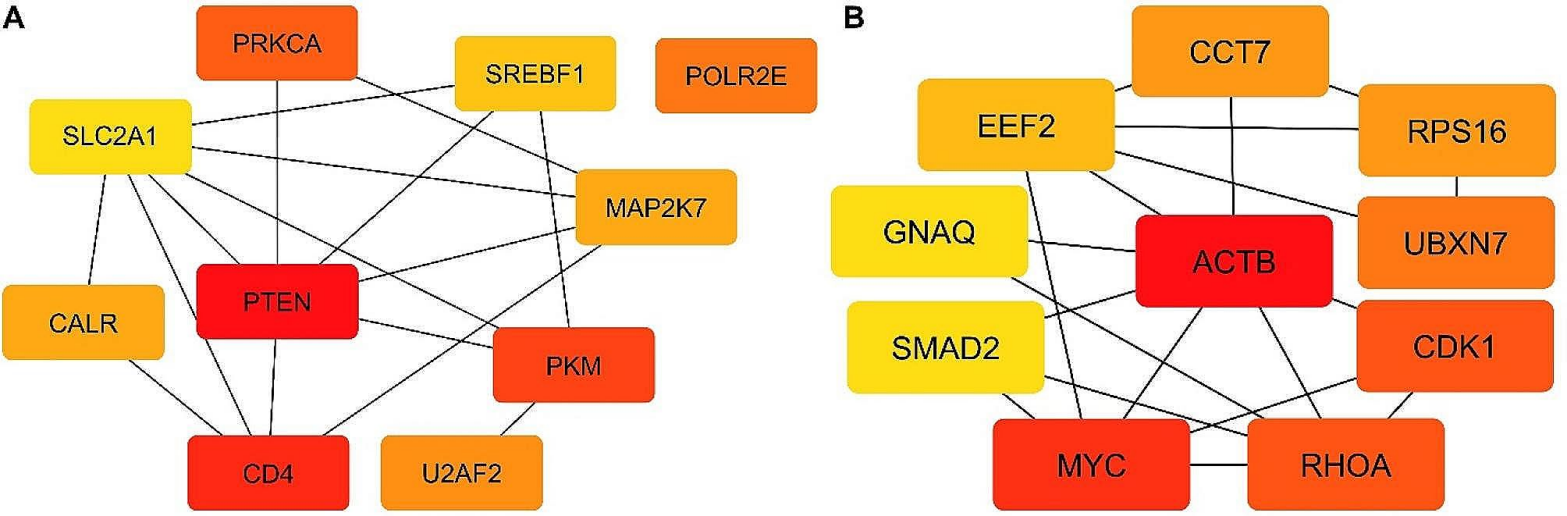
The top 10 hub genes in PPI networks of DE-miRNA target genes. (**A**) Hub I for up-regulated miRNAs. (**B**) Hub II for down-regulated miRNAs. The node color changes gradually from red to yellow in descending order according to the BottleNeck ranking method. *PTEN* and *ACTB* were recognized as key hub genes for up- and down-regulated miRNAs, respectively. The analysis was performed using the cytoHubba plugin of Cytoscape v3.10.0. PPI, protein-protein interaction; DE-miRNA, differentially expressed miRNA

The PPI networks were then filtered using the cytoHubba plugin in Cytoscape v3.10.0 software to identify genes showing the high whole-network connectivity. For up-regulated miRNAs, the top 10 hub genes (Hub I) were Phosphatase and Tensin Homolog (*PTEN*), Protein Kinase C Alpha (*PRKCA*), Pyruvate Kinase M1/2 (*PKM*), CD4 Molecule (*CD4*), RNA Polymerase II, I and III Subunit E (*POLR2E*), Calreticulin (*CALR*), Mitogen-Activated Protein Kinase Kinase 7 (*MAP2K7*), Sterol Regulatory Element Binding Transcription Factor 1 (*SREBF1*), U2 Small Nuclear RNA Auxiliary Factor 2 (*U2AF2*), and Solute Carrier Family 2 Member 1 (*SLC2A1*), as per the decreasing order of degree values (Fig. 5A).

For down-regulated DE-miRNAs, Actin Beta (*ACTB*), MYC proto-oncogene (*MYC*), Ras Homolog Family Member A (*RHOA*), Cyclin Dependent Kinase 1 (*CDK1*), UBX Domain Protein 7 (*UBXN7*), Ribosomal Protein S16 (*RPS16*), Chaperonin Containing TCP1 Subunit 7 (*CCT7*), Eukaryotic Translation Elongation Factor 2 (*EEF2*), SMAD Family Member 2 (*SMAD2*), G Protein Subunit Alpha q (*GNAQ*) were found as the top 10 hub genes (Hub II) (Fig. 5B).

Among the hub gene networks for up- and down-regulated miRNAs, *PTEN* and *ACTB* were designated as key hub genes with the highest node degrees of 72 and 119, respectively. Through the DE-miRNA-hub gene network construction, most of the hub genes were found to be potentially modulated by up-regulated hsa-miR-4505 and hsa-miR-4743-5p and down-regulated hsa-miR-3613-3p and hsa-miR-4668-5p (Table 4).

**Table 4:** The list of hub genes and targeted DE-miRNAs

Hub I		Hub II	
Hub gene	DE-miRNAs targeting hub gene	Hub gene	DE-miRNAs targeting hub gene
*PTEN*	miR-4505	*ACTB*	miR-3613-3p
*PRKCA*	miR-4505	*MYC*	miR-4668-5p
*PKM*	miR-4505	*RHOA*	miR-3613-3p
*CD4*	miR-4505	*CDK1*	miR-3613-3p, miR-4750-3p
*POLR2E*	miR-4505	*UBXN7*	miR-320e, miR-3613-3p, miR-4668-5p
*CALR*	miR-6846-5p	*RPS16*	miR-3613-3p
*MAP2K7*	miR-4505	*CCT7*	miR-320e
*SREBF1*	miR-4743-5p	*EEF2*	miR-7850-5p
*U2AF2*	miR-4505, miR-4743-5p	*SMAD2*	miR-3613-3p, miR-4668-5p
*SLC2A1*	miR-4505	*GNAQ*	miR-3613-3p, miR-4668-5p

DE-miRNA, differentially expressed miRNA; *PTEN*, Phosphatase and Tensin Homolog; *PRKCA*, Protein Kinase C Alpha; *PKM*, Pyruvate Kinase M1/2; *CD4*, CD4 Molecule; *POLR2E*, RNA Polymerase II, I and III Subunit E; *CALR*, Calreticulin; *MAP2K7*, Mitogen-Activated Protein Kinase Kinase 7; *SREBF1*, Sterol Regulatory Element Binding Transcription Factor 1; *U2AF2*, U2 small nuclear RNA auxiliary factor 2; *SLC2A1*, Solute Carrier Family 2 Member 1; *ACTB*, Actin Beta; *MYC*, MYC proto-oncogene; *RHOA*, Ras Homolog Family Member A; *CDK1*, Cyclin Dependent Kinase 1; *UBXN7*, UBX Domain Protein 7; *RPS16*, Ribosomal Protein S16; *CCT7*, Chaperonin Containing TCP1 Subunit 7; *EEF2*, Eukaryotic Translation Elongation Factor 2; *SMAD2*, SMAD Family Member 2; *GNAQ*, G Protein Subunit Alpha q

As listed in Table 5, Hub I genes were significantly enriched in response to stress, insulin, and oxygen-containing compound, as well as regulation of ErbB and hypoxia-inducible factor 1 (HIF-1) signaling pathways. Among the top 10 biological functions of Hub II genes, TGF-β and Hippo signaling pathways, and platelet activation were most associated with the development of diabetic atherosclerosis. Moreover, based on KEGG pathway analysis, several other T2DM-CAD-related processes, including leukocyte transendothelial migration, fluid shear stress and atherosclerosis, focal adhesion, along with apelin, Wnt, and chemokine signaling pathways, were found to enrich Hub II genes (Table 5).

**Table 5:** Functional enrichment analysis of hub gene modules for up-regulated and down-regulated miRNAs in T2DM-CAD

Hub	Category	Term	*p*-Value	Adjusted *p*-Value	Genes
I	GO: BP	Response to stress	1.53 × 10^−6^	0.010	*CD4*,* PKM*,* CALR*,* SREBF1*,* PTEN*,* MAP2K7*,* PRKCA*,* SLC2A1*,* POLR2E*
	GO: BP	Positive regulation of ERK1 and ERK2 cascade	2.72 × 10^−6^	0.012	*CD4*,* PTEN*,* MAP2K7*,* PRKCA*
	GO: BP	Transmembrane receptor protein tyrosine kinase signaling pathway	3.01 × 10^−6^	0.012	*CD4*,* SREBF1*,* PTEN*,* PRKCA*,* POLR2E*
	GO: BP	Response to insulin	4.02 × 10^−6^	0.012	*PKM*,* SREBF1*,* PTEN*,* SLC2A1*
	GO: BP	Positive regulation of macromolecule metabolic process	4.17 × 10^−5^	0.041	*CD4*,* U2AF2*,* CALR*,* SREBF1*,* PTEN*,* MAP2K7*,* PRKCA*,* POLR2E*
	GO: BP	Response to oxygen-containing compound	4.22 × 10^−5^	0.041	*CD4*,* PKM*,* CALR*,* SREBF1*,* PTEN*,* SLC2A1*
	GO: BP	Response to nutrient levels	5.32 × 10^−5^	0.043	*CD4*,* PKM*,* SREBF1*,* SLC2A1*
	GO: MF	Enzyme binding	1.08 × 10^−6^	0.004	*CD4*,* U2AF2*,* CALR*,* SREBF1*,* PTEN*,* MAP2K7*,* PRKCA*,* SLC2A1*
	GO: MF	Protein-containing complex binding	9.87 × 10^−6^	0.016	*CD4*,* PKM*,* CALR*,* SREBF1*,* PTEN*,* PRKCA*
	GO: MF	Kinase binding	1.71 × 10^−5^	0.019	*CD4*,* SREBF1*,* PTEN*,* MAP2K7*,* SLC2A1*
	KEGG	Insulin resistance	2.01 × 10^−5^	0.002	*SREBF1*,* PTEN*,* SLC2A1*
	KEGG	ErbB signaling pathway	8.2 × 10^−4^	0.032	*MAP2K7*,* PRKCA*
	KEGG	Insulin secretion	8.0 × 10^−4^	0.032	*PRKCA*,* SLC2A1*
	KEGG	HIF-1 signaling pathway	0.001	0.032	*PRKCA*,* SLC2A1*
II	GO: BP	Protein deubiquitination	1.53 × 10^−7^	0.002	*SMAD2*,* ACTB*,* CDK1*,* RHOA*,* MYC*
	KEGG	TGF-beta signaling pathway	1.26 × 10^−5^	0.001	*SMAD2*,* RHOA*,* MYC*
	KEGG	Platelet activation	2.95 × 10^−5^	0.002	*GNAQ*,* ACTB*,* RHOA*
	KEGG	Hippo signaling pathway	5.72 × 10^−5^	0.002	*SMAD2*,* ACTB*,* MYC*
	KEGG	Rap1 signaling pathway	1.3 × 10^−4^	0.004	*GNAQ*,* ACTB*,* RHOA*
	KEGG	Leukocyte transendothelial migration	0.001	0.021	*ACTB*,* RHOA*
	KEGG	Fluid shear stress and atherosclerosis	0.002	0.026	*ACTB*,* RHOA*
	KEGG	Apelin signaling pathway	0.002	0.026	*SMAD2*,* GNAQ*
	KEGG	Wnt signaling pathway	0.003	0.031	*RHOA*,* MYC*
	KEGG	Chemokine signaling pathway	0.004	0.038	*GNAQ*,* RHOA*
	KEGG	Focal adhesion	0.004	0.040	*ACTB*,* RHOA*
	Reactome	Deubiquitination	1.5 × 10^−7^	1.6 × 10^−4^	*SMAD2*,* ACTB*,* CDK1*,* RHOA*,* MYC*
	Reactome	Signaling by TGF-beta Receptor Complex	6.36 × 10^−6^	0.004	*SMAD2*,* RHOA*,* MYC*
	Reactome	SMAD2/SMAD3:SMAD4 heterotrimer regulates transcription	1.3 × 10^−4^	0.029	*SMAD2*,* MYC*

T2DM-CAD; type 2 diabetes mellitus with coronary artery disease; GO, Gene Ontology; BP, biological process; MF, molecular function; KEGG, Kyoto Encyclopedia of Genes and Genomes

*p*-value and adjusted *p*-value < 0.05 were statistically significant

### Validation of differentially expressed miRNAs by RT-qPCR

The differential expression of five miRNAs (hsa-miR-3613-3p, hsa-miR-4505, hsa-miR-4668-5p, hsa-miR-4743-5p, hsa-miR-4750-3p) selected from miRNA profiling using microarrays based on the highest FC, AUC value and their potential biological significance in the pathogenesis of CAD in T2DM, was validated by RT-qPCR in a separate larger replication cohort comprising of 94 patients (T2DM with CAD group, *n* = 30; T2DM group, *n* = 30; CAD group, *n* = 16; control group, *n* = 18). The expression levels of miRNAs were normalized to the expression of U6 snRNA as an endogenous control. hsa-miR-4505, hsa-miR-4743-5p, and hsa-miR-4750-3p were constantly detected in all samples, but hsa-miR-3613-3p and hsa-miR-4668-5p could only be detected at very low levels by RT-qPCR, making their valid quantification impossible. Hence, these two miRNAs were excluded from subsequent analysis.

**Fig. 6:**
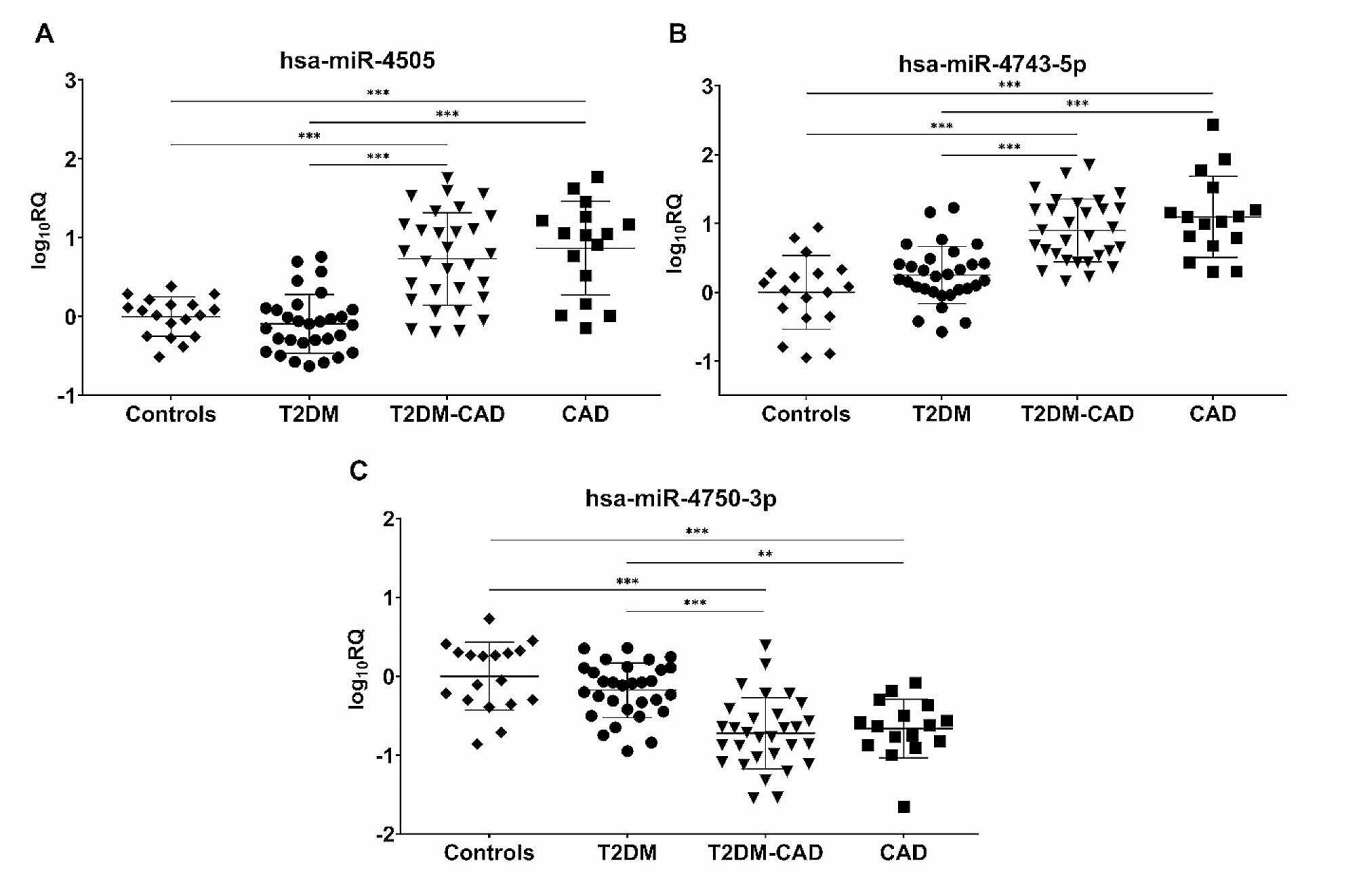
Comparison of miRNA expression in T2DM-CAD, T2DM, CAD and control groups in the validation cohort. The relative expressions of (**A**) hsa-miR-4505; (**B**) hsa-miR-4750-3p; (**C**) hsa-miR-4743-5p were calculated using the 2^−ΔΔCt^ method (with U6 snRNA as an endogenous control) and logarithmically transformed (log_10_RQ). hsa-miR-4505 and hsa-miR-4743-5p were significantly up-regulated, and hsa-miR-4750-3p was down-regulated in T2DM-CAD patients as compared to T2DM subjects and controls. One-way ANOVA with Tukey’s post hoc test for unequal *n* was used to determine the significance of differences between groups. Scatter plots show mean expression levels of miRNAs with standard deviation. Asterisks indicate statistically significant differences. *** *p* < 0.001, ** *p* < 0.01. T2DM-CAD, type 2 diabetes mellitus with coronary artery disease; T2DM, type 2 diabetes mellitus; CAD, coronary artery disease; RQ, relative expression

Similar to the discovery phase of the study, hsa-miR-4505 and hsa-miR-4743-5p were found to be significantly up-regulated, whereas hsa-miR-4750-3p was down-regulated in both T2DM-CAD group compared to the T2DM group and the T2DM-CAD group compared to the control group (all *p* < 0.001) (Fig. 6). The most increased miRNA expression was detected in hsa-miR-4505 with a 6.64-fold change, and a 3.50-fold decrease in expression level was found in hsa-miR-4750-3p in T2DM-CAD patients compared to T2DM individuals.

### Evaluation of the diagnostic value of tested miRNAs in T2DM-CAD

**Fig. 7:**
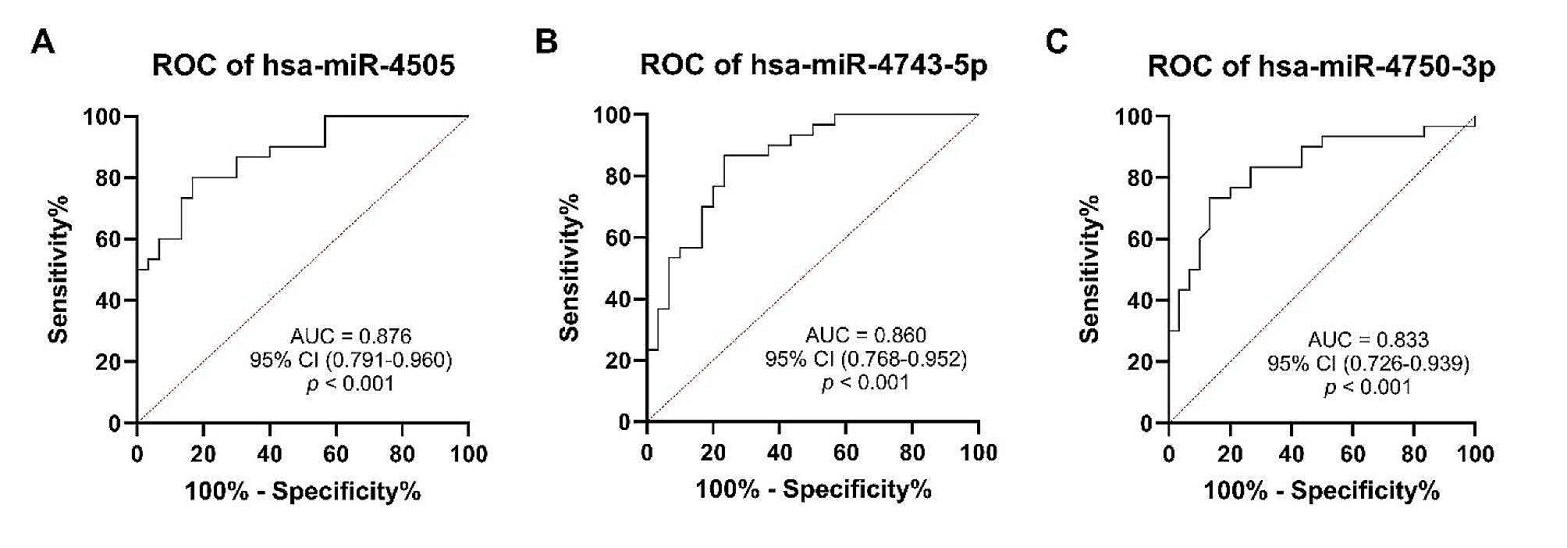
ROC curve analysis of the selected miRNAs in the validation set. (**A**) hsa-miR-4505, (**B**) hsa-miR-4743-5p, and (**C**) hsa-miR-4750-5p yielded good diagnostic potential for CAD in T2DM. The AUC value, 95% CI, and level of statistical significance are indicated in each graph. *p*-value < 0.05 was statistically significant. ROC, receiver operating characteristic; CAD, coronary artery disease; T2DM, type 2 diabetes mellitus; AUC, area under the curve; CI, confidence interval

ROC curve analysis was conducted to assess the diagnostic value of the three identified miRNAs as candidate biomarkers for CAD in T2DM. All three miRNAs, hsa-miR-4505, hsa-miR-4743-5p, and hsa-miR-4750-3p, showed AUCs reaching 0.876, 0.860, and 0.833, respectively, indicating that they possess good diagnostic potential in discriminating between T2DM-CAD and T2DM patients (Fig. 7). In addition, we calculated the Youden Index to determine cut-off values. The optimal cut-off points (log_10_RQ) for hsa-miR-4505, hsa-miR-4743-5p, and hsa-miR-4750-3p were 0.22, 0.43, and − 0.54, respectively. The corresponding sensitivities and specificities to these points were evaluated and are presented in **Additional file 1: Table S8**.

### Identification of the best combination of miRNAs for CAD detection in T2DM

**Fig. 8:**
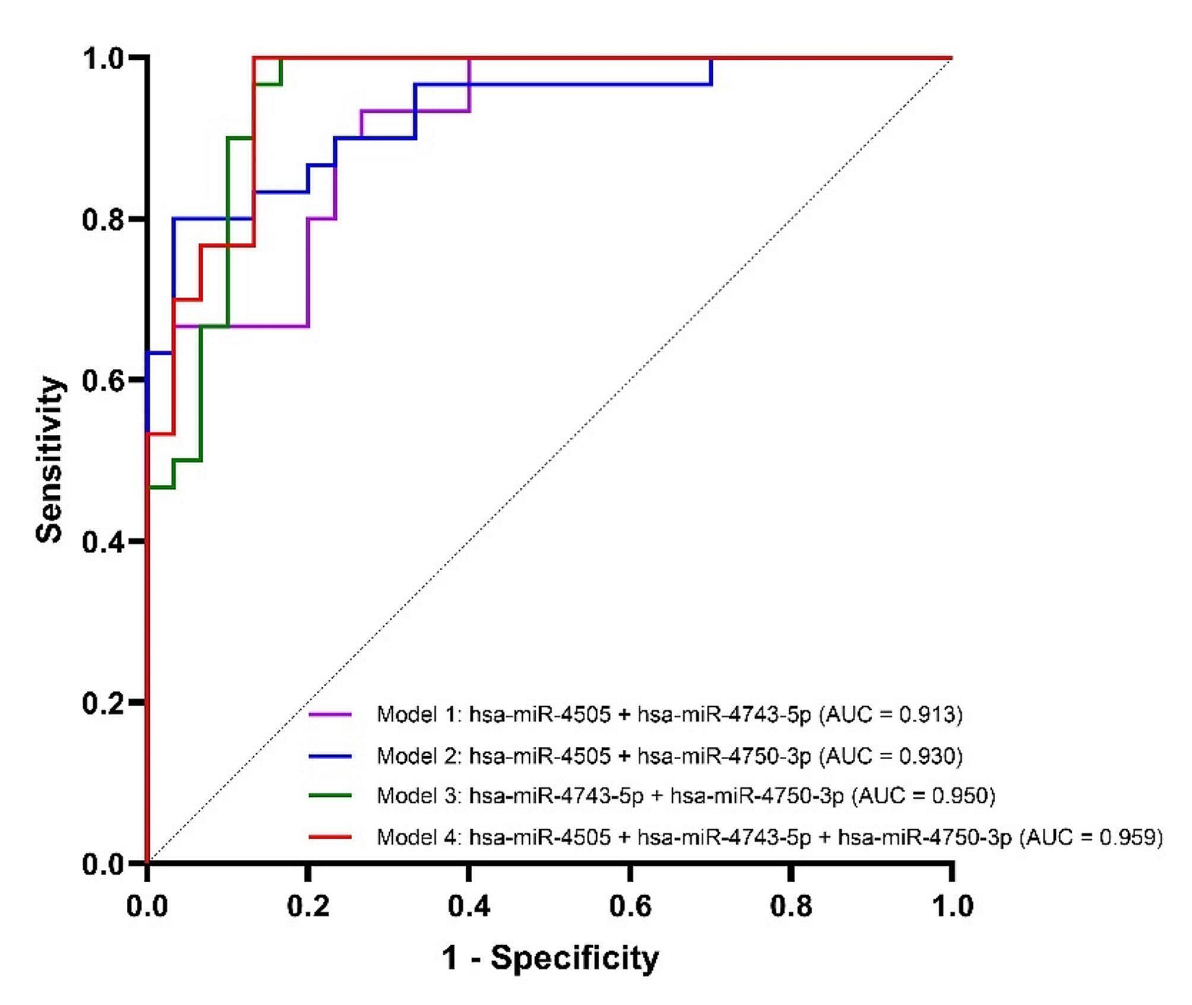
ROC curves for the miRNA-based diagnostic classification models in the validation cohort. The highest AUC (0.959, *p*-value < 0.0001) was obtained for the combination of hsa-miR-4505, hsa-miR-4743-5p, and hsa-miR-4750-3p. ROC, receiver operating characteristic; AUC, area under the curve

To investigate the combination of miRNAs with the strongest classification power for discrimination between T2DM-CAD and T2DM subjects, logistic regression models were developed. Based on the results of the miRNA expression levels obtained during the validation phase of the study, four models were derived: model 1 was built on the expression of hsa-miR-4505 and hsa-miR-4743-5p as independent variables, model 2 included hsa-miR-4505 and hsa-miR-4750-3p, model 3 was based on the expression of hsa-miR-4743-5p and hsa-miR-4750-3p, while model 4 included all three variables. To assess the diagnostic potential of the constructed models, ROC curves were plotted (Fig. 8), and the basic parameters and common quality measures of the models are summarized in **Additional File 1: Table S9**. All miRNA-based signature revealed higher diagnostic values compared to the those for miRNAs used separately. The data indicated that the best classification accuracy for detecting CAD in T2DM was achieved by the three-miRNA panel consisting of hsa-miR-4505, hsa-miR-4743-5p, and hsa-miR-4750-3p (model 4), with 100.00% sensitivity and 86.67% specificity (AUC = 0.959, *p*-value < 0.0001).

## Discussion

T2DM and CAD constitute two closely inter-related clinical entities whose concurrent prevalence is steadily increasing [37]. It is now well established that T2DM accelerates the onset of CAD, however, the pathogenetic molecular mechanisms underlying diabetic atherosclerosis are not fully understood [3]. Despite substantial improvement in the diagnostic strategies in cardiovascular diseases, effective non-invasive screening methods for early detection of CAD in T2DM are still urgently needed [1, 7, 9].

In the present study, we investigated the potential role of circulating miRNAs as novel biomarkers for CAD in patients with T2DM. To find a unique miRNA profile in plasma samples from T2DM-CAD patients, we performed microarray analysis using a broad panel of 2,578 human miRNAs that have not been previously assessed in coexisting CAD and T2DM. Our results revealed that the expression of twelve miRNAs was significantly altered in T2DM-CAD patients compared to T2DM subjects. Moreover, upon the series of bioinformatic analyses, we uncovered the potential biological significance of DE-miRNA target genes in the pathogenesis of diabetic atherosclerosis. To the best of our knowledge, we have demonstrated for the first time a specific three-miRNA model based on the combined expression of hsa-miR-4505, hsa-miR-4743-5p, and hsa-miR-4750-3p that can effectively differentiate between T2DM patients with and without CAD.

Based on the miRNA expression profiling with microarray platform, we detected a significant increase in the expression levels of three miRNAs (hsa-miR-4505, hsa-miR-4743-5p, hsa-miR-6846-5p), while nine miRNAs (hsa-miR-3613-3p, hsa-miR-4668-5p, hsa-miR-4706, hsa-miR-6511b-5p, hsa-miR-6750-5p, hsa-miR-4750-3p, hsa-miR-320e, hsa-miR-4717-3p, hsa-miR-7850-5p) were down-regulated in T2DM-CAD patients compared to T2DM individuals. Among them, hsa-miR-4505 and hsa-miR-3613-3p appeared to be the most dysregulated miRNAs in T2DM-CAD. So far, only limited studies have exploited the large-scale methods to provide comprehensive analysis of miRNA patterns in patients with T2DM-CAD [38–40]. Leveraging the NanoString nCounter technology, Bielska et al. found that six miRNAs (miR-615-3p, miR-3147, miR-1224-5p, miR-5196-3p, miR-6732-3p, and miR-548b-3p) were overexpressed in the serum of T2DM patients with ischemic heart disease as compared to those with uncomplicated T2DM [38]. In turn, Zhang et al. uncovered a set of 138 extracellular vesicle-carried DE-miRNAs in T2DM-CAD patients by using small RNA sequencing [39]. Nevertheless, these miRNAs have only been evaluated for their utility in distinguishing between T2DM-CAD patients and healthy controls [39]. Similar to prior reports, most of the identified miRNAs in our study, with the exception of hsa-miR-4505, hsa-miR-3613-3p and hsa-miR-320e, have not been previously investigated in CAD and/or T2DM and appear to be novel in the genomic control of T2DM complicated by CAD.

To date, it has been shown that miR-4505 was down-regulated in patients with acute myocardial infarction, whereas miR-3613-3p and miR-320e were up-regulated in patients with stable CAD, but the pattern of changes was not consistent with those obtained in our study [41–43]. These discrepancies in miRNA expression levels can be partially explained by the different sample types (plasma, serum, exosomes), the analytical techniques utilized for miRNA profiling, and differences in ethnicity and disease stages among the study populations. Nonetheless, similar to the current observation, a down-regulation of the 5′-isomiR of miR-4717 was noticed in patients affected by acute myocardial infarction [44]. Importantly, plasma miR-4717-5p was found to distinguish patients with ST-segment elevation and non-ST-segment elevation myocardial infarction from controls with AUCs above 0.800, supporting its potential as a diagnostic biomarker for CAD [44].

Functional annotation analyses of our DE-miRNAs revealed an overrepresentation of genes in terms related to organelle, cellular nitrogen compound metabolic process, biosynthetic process, and ion binding. In addition, the targets of hsa-miR-4505, hsa-miR-4743-5p and hsa-miR-4750-3p were significantly enriched in pathways relevant to the initiation of atherogenesis, including fatty acid metabolism, the neurotrophin signaling pathway, and leukocyte transendothelial migration.

Metabolism of fatty acids engages several inter-connected processes, including fatty acid biosynthesis and degradation, which we found to be modulated by hsa-miR-4505, hsa-miR-4743-5p and hsa-miR-4750-3p [45]. Disturbed fatty acid metabolism has been implicated in diabetes, as evidenced by elevated levels of circulating triglyceride-rich lipoproteins, saturated fatty acids, and increased fatty acid oxidation [46]. Importantly, endothelial cells exposed to hyperglycemia and insulin resistance can rapidly switch from aerobic glycolysis as the predominant source of energy towards enhanced fatty acid β-oxidation [46, 47]. Since a significant amount of NADH is generated, the disturbances in redox homeostasis and increased production of harmful reactive oxygen species (ROS) occur [48, 49]. Moreover, excess free fatty acids lead to activation of protein kinase C (PKC), the nuclear factor-kappa B (NF-κB) pathway, and an increase in endothelial activation markers such as intercellular adhesion molecule-1 (ICAM-1) and vascular cell adhesion molecule-1 (VCAM-1) [50]. Thus, the dysregulation of fatty acid metabolism triggers a myriad of detrimental effects, aggravating oxidative stress, endothelial permeability and inflammation, ultimately contributing to endothelial injury and atherogenesis [50].

In a recent study, and in agreement with our microarray data, up-regulation of miR-4505 was found to exacerbate endothelial injury, suggesting its potential as a novel proatherogenic molecular target [51]. NF-κB signaling activated in response to pro-inflammatory stimuli in endothelial cells has been shown to induce miR-4505 expression and, by targeting heat shock protein A12B, enhance endothelial permeability by reducing cell-cell junctions, especially vascular endothelial (VE)-cadherin [51]. Similarly, Liu et al. confirmed the atheroprone effect of miR-3613-3p down-regulation in heat-stressed endothelial cells, identifying the mitogen-activated protein kinase kinase kinase 2 (MAP3K2)/p38/caspase-3 pathway as its downstream target [52–54]. As previously reported, we reinforced that hsa-miR-3613-3p can modulate the Hippo signaling pathway, consisting of a cascade of kinases, transcriptional coactivators and critical effectors such as Yes-associated protein (YAP) and transcriptional coactivator with PDZ-binding motif (TAZ) [55]. A variety of stress signals, including hypoxia, endoplasmic reticulum stress or heat stress, can trigger the Hippo signaling pathway [55]. Interestingly, inactivation of Hippo/YAP signaling has been found to promote endothelial inflammation in a model of diabetes-accelerated atherosclerosis by enhancing monocyte-endothelial cell adhesion and production of pro-inflammatory cytokines [56]. Additionally, Hu et al. stated that inhibition of the Hippo-YAP/TAZ/miR-496 pathway in oxidized-LDL (ox-LDL) treated endothelial cells leads to their dysfunction [57]. Therefore, the exact role of the miR-3613-3p/Hippo signaling pathway in the pathogenesis of diabetic atherosclerosis needs to be clarified.

A comprehensive bioinformatic analysis highlighted that pathways in cancers, calcium ion binding, cell-to-cell communication, and actin cytoskeleton dynamics were among the most enriched terms for the set of up-regulated miRNAs in our study. It is well recognized, that under hyperglycemic milieu, the inflamed endothelium changes its structural and functional properties, mainly through RhoA, a small Rho GTPase [58]. Mechanistically, chemokine-induced activation of these intracellular signaling proteins increases cytoskeletal dynamics and contractility, and leukocyte polarization, enabling directional binding of endothelial adhesion molecules to their respective ligands on the leukocyte surfaces [59]. Finally, calcium-dependent VE-cadherin-based adherens junctions are disrupted, disassembled, internalized and ultimately degraded, enhancing endothelial permeability and promoting leukocyte transendothelial migration, a key step in atherosclerotic plaque formation [58, 60]. Hence, we proposed hsa-miR-4505, hsa-miR-4743-5p and hsa-miR-6846-5p as novel potential atheromiRs, but in vivo and in vitro studies are needed to further investigate the role of these miRNAs in diabetic atherosclerosis.

Among the DE-miRNAs identified in T2DM-CAD, we discovered hsa-miR-4505, hsa-miR-4717-3p, and hsa-miR-7850-5p to be essential regulators of signaling by neurotrophins, predominantly NGF, through TRK receptors, which are highly expressed in endothelial cells and VSMCs [61]. Emerging evidence indicates that the expression of NGF dramatically increases in response to vascular injury, providing the activation of several pathways, including MAPK/extracellular signal-regulated kinase (ERK), phosphatidylinositol 3-kinase (PI3K)-Akt, and the small GTPase RhoA, the latter of which promotes VSMC proliferation and migration [61]. In contrast, Chaldakov et al. showed decreased levels of NGF in plasma samples from patients with metabolic syndrome and in the atherosclerotic coronary vessel wall, especially those with advanced lesions, confirming its involvement in the evolution of atherosclerotic plaques in T2DM [62, 63].

**Fig. 9:**
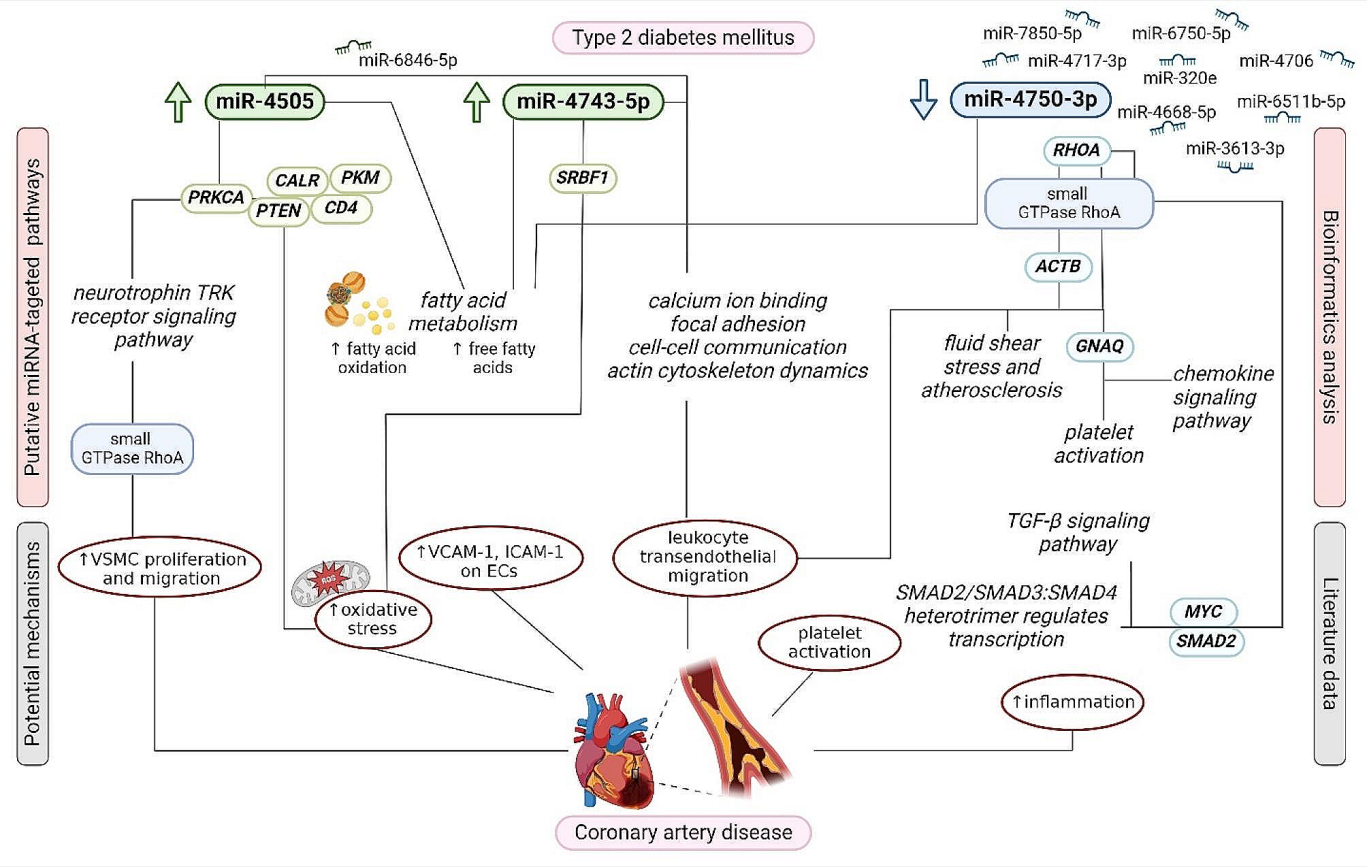
The schematic diagram of potential miRNA-gene-pathway axes involved in the development of CAD in T2DM. Created with BioRender.com, accessed on 28 May 2024. CAD, coronary artery disease; T2DM, type 2 diabetes mellitus; *PRKCA*, Protein Kinase C Alpha; *CALR*, Calreticulin; *PTEN*, Phosphatase and Tensin Homolog; *PKM*, Pyruvate Kinase M1/2; *CD4*, CD4 Molecule; *SRBF1*, Sterol Regulatory Element Binding Transcription Factor 1; *RHOA*, Ras Homolog Family Member A; *ACTB*, Actin Beta; *GNAQ*, G Protein Subunit Alpha q; *MYC*, MYC proto-oncogene; *SMAD2*, SMAD Family Member 2; TRK, tropomyosin kinase receptor; VSMC, vascular smooth muscle cell; VCAM-1, vascular cell adhesion molecule-1; ICAM-1, intercellular adhesion molecule-1; EC, endothelial cell; TGF-β, transforming growth factor-β

As newly emerging epigenetic regulators, miRNAs could be involved in the specific control of genes contributing to the development of diabetic atherosclerosis [14]. By the PPI network analysis, we extracted the top ten hub genes separately for up-regulated (*PTEN*,* PRKCA*,* PKM*,* CD4*,* POLR2E*,* CALR*,* MAP2K7*,* SREBF1*,* U2AF2*,* SLC2A1*) and down-regulated (*ACTB*,* MYC*,* RHOA*,* CDK1*,* UBXN7*,* RPS16*,* CCT7*,* EEF2*,* SMAD2*,* GNAQ*) miRNAs in T2DM-CAD. *PTEN* and *ACTB* were recognized as the top guide genes for up- and down-regulated miRNAs, respectively (Fig. 9).

*PTEN* acts as a stress-balancing molecule and is involved in the regulation of endoplasmic reticulum stress, endothelial cell apoptosis, inflammation, and VSMC proliferation and migration in response to ox-LDL-induced oxidative stress [64–69]. Moreover, calcium-dependent and diacylglycerol-activated PKCα (*PRKCA*) under hyperglycemic settings has been found to promote ROS-producing enzymes and oxidative stress, the expression of endothelial adhesion molecules, pro-inflammatory cytokine production, and deteriorate nitric oxide-dependent vasodilation, indicating that it may be a mediator of the onset of diabetes-related atherosclerosis [70]. Similarly, *CALR*, encoding calreticulin, an endoplasmic reticulum stress marker, and MAP kinase kinase 7 (*MAP2K7*), a key upstream transducer of stress-activated protein kinase, have been evidenced as vital contributors to endothelial dysfunction in an oxidative stress environment [71, 72]. In line with previous reports, the results of our functional enrichment analysis of *PTEN*, *PRKCA*, *CALR*, *MAP2K7* argue for their involvement mainly in the regulation of the oxidative stress response in diabetes. Additionally, recent studies have suggested that monocytes and macrophages from patients with atherosclerotic CAD overuse glucose and overexpress several glycolysis-related genes, including *PKM* and *SLC2A1*, encoding two PKM isoforms (PKM1 and PKM2) and glucose transporter 1 (GLUT-1), respectively [73, 74]. PKM2 and GLUT-1 have been demonstrated to promote the aberrant differentiation of macrophages towards a pro-inflammatory M1 phenotype, enhance ox-LDL uptake, thereby fostering foam cell formation and exacerbating inflammation [73–77]. *SREBF1*, another identified hub gene in our study, plays a central role in regulating lipid biosynthesis and uptake, and its elevated levels were found during the progression of foam cell formation in ox-LDL-treated macrophages [78]. Hence, *PKM*, *SLC2A1* along with *SREBF1* bridge metabolic and inflammatory dysfunctions in the pathogenesis of diabetic atherosclerosis. Fu et al. revealed the levels of *CD4*, which encodes a glycoprotein expressed on immune cells such as CD4 + T cells, are increased in atherosclerotic plaques, particularly those with advanced lesions [79]. It is of note that *CD4* effectively distinguished T2DM-CAD patients from controls [79].

As we mentioned above, β-actin, encoded by *ACTB*, is a core cytoskeleton protein that, as an effector of the small GTPase RhoA, regulates cell structure, proliferation and migration, contributing to vascular endothelial dysfunction and remodeling [80]. The elevated expression of *ACTB* and *MYC* was also detected in epicardial adipose tissue samples from patients with CAD, implying their paracrine modulatory role in the development of atherosclerosis [81]. Interestingly, activation of *MYC*, a key downstream target gene of the Wnt signaling pathway, has been observed at an early stage of atherosclerotic plaque evolution [82]. Similarly, up-regulation of RhoA in a hyperglycemic state aggravates endothelial dysfunction and increases endothelial permeability through stress fiber formation, focal adhesion and cell contraction [83, 84]. Conversely, inhibition of RhoA, which is a downstream effector of Hippo/YAP/TAZ activation, exerts atheroprotective and anti-inflammatory effects [85]. *MYC*, *RHOA*, *SMAD2*, known as TGF-β transducers, along with *CDK1*, have been identified as stimulators of VSMC proliferation, migration, apoptosis or ferroptosis, thereby exacerbating diabetic atherosclerosis [84, 86–89]. Deng et al. and Yang et al. postulated that disturbed fluid shear stress activates TGF-β receptors, amplifies SMAD2/SMAD3 activation, and thus the expression of pro-inflammatory genes [90, 91]. These observations may partially explain the increased expression of *SMAD2* in endothelial cells derived from patients with CAD [92]. In accordance with previous studies, our analysis of KEGG pathways confirmed the involvement of *GNAQ* in platelet activation [93]. Notably, all of the identified GO terms, KEGG and Reactome pathways for hub genes of down-regulated miRNAs are strongly related to the development and progression of diabetic atherosclerosis.

To verify the robustness of the microarray analysis, we further validated the expression of five selected DE-miRNAs using RT-qPCR in a larger independent cohort, confirming the overexpression of hsa-miR-4505, hsa-miR-4743-5p and the down-regulation of hsa-miR-4750-3p in T2DM-CAD patients with respect to T2DM individuals. Nevertheless, the expression of the highly down-regulated hsa-miR-3613-3p and hsa-miR-4668-5p could only be detected at very low levels, making their valid quantification impossible. This lack of concurrence between these two methods may be partially elucidated by the observed lower correlation for down-regulated genes in prior investigations due to the greater variability associated with decreased reaction efficiencies found at later cycles, where genes with low expression levels respond [94].

Evaluating the diagnostic accuracy of hsa-miR-4505, hsa-miR-4743-5p and hsa-miR-4750-3p, we showed that each of these miRNAs could be a good stand-alone discriminator of CAD in T2DM patients. These results are consistent with prior reports that have demonstrated comparable performance values for different single miRNAs in CAD associated with T2DM [15–18, 95, 96]. Innovatively, we displayed that the model based on combining the expression of hsa-miR-4505, hsa-miR-4743-5p, hsa-miR-4750-3p yielded the highest diagnostic accuracy with an excellent AUC of 0.959 (95% CI, 0.914–1.000; *p* < 0.0001) and 100.00% sensitivity and 86.67% specificity in comparison with each miRNA individually and constructed panels of two miRNAs in detecting CAD in T2DM patients. Likewise, other authors suggested that the two miRNA signatures consisting of miR-3147, miR-615-3p and miR-9, miR-370, respectively, revealed better diagnostic value than these miRNAs considered individually in identifying T2DM patients at high risk of CAD [38, 97]. Although Al-Muhtaresh et al. also achieved good discriminatory efficacy for the combined miR-1 and miR-133 signature (AUC 0.752), this was lower than that for miR-1 alone (AUC 0.802), possibly due to only a marginally statistically significant AUC value for miR-133 [98]. These findings provide the groundwork for future investigations, whereby a panel of miRNAs appears to be more beneficial in predicting the development of CAD in patients with T2DM rather than single miRNAs.

### Limitations of the study

The present study also has some limitations. Firstly, due to the observational nature of the study, the potential impact of residual confounding factors cannot be ruled out. Nevertheless, our investigation was based on the rigorous methodological design and comprehensive data collection methods. Secondly, the sample lacked ethnic diversity, which limits the generalizability of the findings. Thirdly, although functional and pathway enrichment analyzes were performed to determine the potential biological significance of DE-miRNAs, further studies in in vitro and in vivo experimental models are needed to conclusively define the regulatory role of miRNA target genes in the pathogenesis of CAD in T2DM. Fourthly, the lack of inter-laboratory standardization of miRNA detection and the costs associated with miRNA analysis may partially restrict the translation of miRNA-based biomarkers into clinical practice. Finally, the sample size is limited, thus the results should be considered preliminary and verified in large-scale, multi-center studies to confirm the applicability of the three-miRNA signature as a diagnostic biomarker for CAD in patients with T2DM in the general population.

## Conclusions

In summary, the present study revealed a unique profile of circulating plasma-derived miRNAs in T2DM patients with comorbid CAD using microarray technology. Integrated bioinformatics analysis showed that DE-miRNA target genes were mainly enriched in pathways related to regulation of cellular processes, gene expression, fatty acid metabolism, oxidative stress response, neurotrophin signaling pathway, and leukocyte transendothelial migration, known to be involved in the initiation of diabetic atherosclerosis. We independently confirmed that hsa-miR-4505 and hsa-miR-4743-5p are significantly overexpressed, while hsa-miR-4750-3p is down-regulated in T2DM-CAD patients compared to T2DM subjects and may serve as novel non-invasive molecular markers of diabetes-accelerated CAD. Innovatively, we developed a three-miRNA model based on the combined expression of hsa-miR-4505, hsa-miR-4743-5p, hsa-miR-4750-3p, which could effectively detect CAD in T2DM in the Caucasian population, better than each miRNA separately.

### Electronic supplementary material

Supplementary Material 1. Additional file 1: Table S1. Significantly dysregulated miRNAs between the T2DM-CAD and T2DM groups. Table S2. Significantly dysregulated miRNAs between the T2DM-CAD group and the control group. Table S3. Significantly dysregulated miRNAs between the T2DM group and the control group. Table S4. Significantly dysregulated miRNAs between the T2DM-CAD and CAD groups. Figure S1. Venn diagram showing unique and common DE-miRNAs in T2DM-CAD, T2DM, CAD and control groups. Figure S2. Differential miRNA expression in the plasma of T2DM-CAD and T2DM patients. Table S5. Tested miRNAs and their target genes. Table S6. Functional enrichment analysis of the six top-scored clusters for up-regulated miRNA target genes in T2DM-CAD. Table S7. Functional enrichment analysis of the six top-scored clusters for down-regulated miRNA target genes in T2DM-CAD. Table S8. Summary of ROC analysis for testing the diagnostic performance of miRNAs as biomarkers for T2DM-CAD. Table S9. Summary of basic parameters and standard quality measures of miRNA-based models

## Data Availability

The datasets used and/or analyzed during the current study are available from the corresponding author on reasonable request.

## Abbreviations

ANOVA: One-way analysis of variance
AUC: Area under the curve
BMI: Body mass index
BP: Biological Process
CAD: Coronary artery disease
CC: Cellular Component
CI: Confidence interval
Ct: Cycle threshold
DBP: Diastolic blood pressure
DE-miRNA: Differentially expressed miRNA
EGFR: Epidermal growth factor receptor
eGFR: Estimated glomerular filtration rate
FC: Fold change
FDR: False discovery rate
GO: Gene Ontology
HbA1c: Glycated hemoglobin A1c
HDL-C: High-density lipoprotein cholesterol
HIF-1: Hypoxia-inducible factor 1
hs-CRP: High-sensitivity C-reactive protein
ICAM-1: Intercellular adhesion molecule-1
IQR: Interquartile range
KEGG: Kyoto Encyclopedia of Genes and Genomes
LDL-C: Low-density lipoprotein cholesterol
MAPK: Mitogen-activated protein kinases
MCODE: Molecular Complex Detection
MDRD: Modification of Diet in Renal Disease
miRNA, miR: microRNA
MF: Molecular Function
NGF: Nerve growth factor
NF-κB: Nuclear factor-kappa B
ox-LDL: oxidized-low density lipoprotein
PKC: Protein kinase C
PPI: Protein-protein interaction
ROC: Receiver operating characteristic
ROS: Reactive oxygen species
RQ: Relative expression
RT-qPCR: Reverse transcription quantitative real-time polymerase chain reaction
SBP: Systolic blood pressure
SD: Standard deviation
snRNA: Small nuclear RNA
T2DM: Type 2 diabetes mellitus
T2DM-CAD: Type 2 diabetes mellitus with coronary artery disease
TAZ: Transcriptional coactivator with PDZ-binding motif
TC: Total cholesterol
TG: Triglyceride
TGF-β: Transforming growth factor-β
TRK: Tropomyosin kinase
VCAM-1: Vascular cell adhesion molecule-1
VSMC: Vascular smooth muscle cell
WHR: Waist-to-hip ratio
YAP: Yes-associated protein
