# Supplementary material for: Identification of plasma miR-4505, miR-4743-5p and miR-4750-3p as novel diagnostic biomarkers for coronary artery disease in patients with type 2 diabetes mellitus: a case-control study
Source: Cardiovasc Diabetol. 2024 Jul 29;23:278. doi: 10.1186/s12933-024-02374-0 (PMC11287982; doi:10.1186/s12933-024-02374-0)
Supplement: Supplementary file 1 — Supplementary Material 1. Additional file 1: Table S1. Significantly dysregulated miRNAs between the T2DM-CAD and T2DM groups. Table S2. Significantly dysregulated miRNAs between the T2DM-CAD group and the control group. Table S3. Significantly dysregulated miRNAs between the T2DM group and the control group. Table S4. Significantly dysregulated miRNAs between the T2DM-CAD and CAD groups. Figure S1. Venn diagram showing unique and common DE-miRNAs in T2DM-CAD, T2DM, CAD and control groups. Figure S2. Differential miRNA expression in the plasma of T2DM-CAD and T2DM patients. Table S5. Tested miRNAs and their target genes. Table S6. Functional enrichment analysis of the six top-scored clusters for up-regulated miRNA target genes in T2DM-CAD. Table S7. Functional enrichment analysis of the six top-scored clusters for down-regulated miRNA target genes in T2DM-CAD. Table S8. Summary of ROC analysis for testing the diagnostic performance of miRNAs as biomarkers for T2DM-CAD. Table S9. Summary of basic parameters and standard quality measures of miRNA-based models [file 12933_2024_2374_MOESM1_ESM.docx]

***Supplementary Material***

**Identification of plasma miR-4505, miR-4743-5p and miR-4750-3p as novel diagnostic biomarkers for coronary artery disease in patients with type 2 diabetes mellitus: a case-control study**

Joanna Szydełko ^1*^, Marcin Czop ^2^, Alicja Petniak ^2^, Monika Lenart-Lipińska ^1^, Janusz Kocki ^2^, Tomasz Zapolski ^3^ and Beata Matyjaszek-Matuszek ^1^

^1^ Department of Endocrinology, Diabetology and Metabolic Diseases; Medical University of Lublin, Jaczewskiego 8, 20-090 Lublin, Poland

^2^ Department of Clinical Genetics, Medical University of Lublin, Radziwillowska 11, 20-080 Lublin, Poland

^3^ Department of Cardiology, Medical University of Lublin, Jaczewskiego 8, 20-090 Lublin, Poland

^*^ Correspondence: jszydelko@interia.pl: +48-81-72-44-668

Supplementary information content list:

**Supplementary Table 1**. Significantly dysregulated miRNAs between the T2DM-CAD and T2DM groups.

**Supplementary Table 2**. Significantly dysregulated miRNAs between the T2DM-CAD group and the control group.

**Supplementary Table 3.** Significantly dysregulated miRNAs between the T2DM group and the control group.

**Supplementary Table 4.** Significantly dysregulated miRNAs between the T2DM-CAD and CAD groups.

**Supplementary Figure 1**. Venn diagram showing unique and common DE-miRNAs in T2DM-CAD, T2DM, CAD and control groups.

**Supplementary Figure 2.** Differential miRNA expression in the plasma of T2DM-CAD and T2DM patients.

**Supplementary Table 5.** Tested miRNAs and their target genes.

**Supplementary Table 6.** Functional enrichment analysis of the six top-scored clusters for up-regulated miRNA target genes in T2DM-CAD.

**Supplementary Table 7.** Functional enrichment analysis of the six top-scored clusters for down-regulated miRNA target genes in T2DM-CAD.

**Supplementary Table 8.** Summary of ROC analysis for testing the diagnostic performance of miRNAs as biomarkers for T2DM-CAD.

**Supplementary Table 9.** Summary of basic parameters and standard quality measures of miRNA-based models.

**Statements:**

All the raw data supporting the findings in this work can be obtained on request from the corresponding author.

**Supplementary Table 1**. Significantly dysregulated miRNAs between the T2DM-CAD and T2DM groups.

| **miRNA** | **T2DM-CAD** | | **T2DM** | | **FC** | ***p*-Value** | **FDR** |
| --- | --- | --- | --- | --- | --- | --- | --- |
|  | ***n* = 12** | | ***n* = 12** | |  |  |  |
| hsa-miR-4505 | 8.15 ± 2.74 | 7.13 (6.15–10.90) | 2.55 ± 2.74 | 1.22 (0.96–3.83) | 118.00 | < 0.001 ^1^ | < 0.001 |
| hsa-miR-4743-5p | 4.11 ± 2.45 | 3.68 (1.63–6.61) | 1.73 ± 1.80 | 1.37 (0.74–1.82) | 6.85 | 0.014 ^1^ | 0.02 |
| hsa-miR-6846-5p | 2.99 ± 1.66 | 2.00 (1.66–4.92) | 1.62 ± 1.36 | 1.14 (0.90–1.76) | 1.95 | 0.008 ^1^ | 0.01 |
| hsa-miR-602 | 1.53 ± 0.33 | 1.49 (1.25–1.77) | 2.11 ± 0.56 | 2.09 (1.63–2.69) | - 1.52 | 0.005 ^2^ | 0.01 |
| hsa-miR-4539 | 1.14 ± 0.38 | 1.00 (0.92–1.31) | 1.63 ± 0.49 | 1.79 (1.20–1.88) | - 1.56 | 0.011 ^2^ | 0.02 |
| hsa-miR-7850-5p | 0.77 ± 0.21 | 0.76 (0.62–0.88) | 1.45 ± 0.36 | 1.50 (1.15–1.74) | - 1.66 | < 0.001 ^2^ | < 0.001 |
| hsa-miR-6763-5p | 0.94 ± 0.49 | 0.69 (0.58–1.33) | 1.55 ± 0.51 | 1.43 (1.25–1.82) | - 1.67 | 0.010 ^1^ | 0.02 |
| hsa-miR-4717-3p | 1.05 ± 0.45 | 1.07 (0.74–1.43) | 1.92 ± 0.49 | 1.92 (1.54–2.31) | - 1.79 | < 0.001 ^2^ | < 0.001 |
| hsa-miR-320e | 6.05 ± 0.58 | 6.02 (5.73–6.47) | 6.57 ± 0.74 | 6.88 (6.11–7.02) | - 1.83 | 0.039 ^1^ | 0.04 |
| hsa-miR-4722-3p | 0.88 ± 0.35 | 0.76 (0.65–1.16) | 1.95 ± 0.98 | 1.66 (1.32–3.11) | - 1.98 | 0.002 ^2^ | 0.01 |
| hsa-miR-4750-3p | 1.35 ± 0.37 | 1.18 (1.08–1.62) | 2.28 ± 0.78 | 2.19 (1.66–2.68) | - 2.02 | 0.001 ^1^ | < 0.01 |
| hsa-miR-8075 | 6.15 ± 0.75 | 6.22 (5.66–6.62) | 6.95 ± 0.99 | 7.20 (6.29–7.76) | - 2.04 | 0.033 ^1^ | 0.04 |
| hsa-miR-6750-5p | 2.49 ± 0.64 | 2.52 (1.98–2.96) | 3.50 ± 0.71 | 3.58 (2.99–4.03) | - 2.06 | 0.001 ^2^ | < 0.01 |
| hsa-miR-2277-5p | 2.18 ± 0.75 | 1.98 (1.60–2.77) | 3.20 ± 1.13 | 3.06 (2.24–4.20) | - 2.12 | 0.017 ^2^ | 0.02 |
| hsa-miR-6511b-5p | 2.37 ± 0.90 | 2.44 (1.59–3.11) | 3.87 ± 1.02 | 3.58 (2.99–4.77) | - 2.50 | 0.001 ^2^ | < 0.01 |
| hsa-miR-4706 | 2.85 ± 0.74 | 2.73 (2.32–3.29) | 4.06 ± 0.99 | 4.23 (3.35–4.91) | - 2.67 | 0.003 ^2^ | 0.01 |
| hsa-miR-4668-5p | 2.91 ± 2.27 | 1.68 (1.13–4.58) | 6.16 ± 3.05 | 7.17 (4.29–8.28) | - 66.16 | 0.017 ^1^ | 0.02 |
| hsa-miR-3613-3p | 2.71 ± 2.46 | 1.41 (0.84–4.28) | 6.66 ± 3.23 | 7.79 (4.13–9.03) | - 103.27 | 0.002 ^1^ | 0.01 |

Values are presented as mean ± SD and median (interquartile range, IQR). ^1^ Mann–Whitney *U* test; ^2^ Student’s *t*-test. *p*-value < 0.05 and FDR ≤ 0.05 were statistically significant. FC > 1.5 or < -1.5.

T2DM-CAD, type 2 diabetes mellitus with coronary artery disease; T2DM, type 2 diabetes mellitus; FC, fold change; FDR, false discovery rate

**Supplementary Table 2.** Significantly dysregulated miRNAs between the T2DM-CAD group and the control group.

| **miRNA** | **T2DM-CAD** | | **Controls** | | **FC** | ***p*-Value** | **FDR** |
| --- | --- | --- | --- | --- | --- | --- | --- |
|  | ***n* = 12** | | ***n* = 6** | |  |  |  |
| hsa-miR-4505 | 8.15 ± 2.74 | 7.13 (6.15–10.90) | 1.08 ± 0.55 | 1.10 (0.58–1.41) | 119.14 | < 0.001 ^2^ | < 0.001 |
| hsa-miR-4743-5p | 4.11 ± 2.45 | 3.68 (1.63–6.61) | 0.94 ± 0.16 | 0.94 (0.89–0.96) | 8.54 | 0.007 ^2^ | 0.02 |
| hsa-miR-320a | 4.14 ± 1.92 | 4.48 (2.19–5.91) | 2.01 ± 1.60 | 1.70 (0.68–2.57) | 5.97 | 0.033 ^2^ | 0.05 |
| hsa-miR-6789-5p | 3.98 ± 1.35 | 4.38 (2.81–5.09) | 2.20 ± 1.11 | 1.93 (1.43–2.30) | 5.83 | 0.013 ^2^ | 0.03 |
| hsa-miR-4687-3p | 4.08 ± 1.33 | 4.63 (3.10–5.02) | 2.55 ± 1.60 | 2.39 (1.51–3.01) | 4.79 | 0.046 ^2^ | 0.05 |
| hsa-miR-320b | 3.26 ± 1.87 | 2.84 (1.53–5.17) | 1.29 ± 0.74 | 1.09 (0.85–1.36) | 4.34 | 0.026 ^2^ | 0.04 |
| hsa-miR-4734 | 3.13 ± 1.33 | 3.51 (2.25–4.17) | 1.63 ± 1.21 | 1.25 (0.80–2.26) | 4.05 | 0.034 ^2^ | 0.05 |
| hsa-miR-6858-5p | 3.40 ± 1.06 | 3.55 (2.88–4.29) | 2.26 ± 1.02 | 1.80 (1.69–3.03) | 3.52 | 0.044 ^2^ | 0.05 |
| hsa-miR-6068 | 2.71 ± 1.54 | 2.63 (1.27–4.09) | 1.08 ± 0.63 | 0.94 (0.81–1.04) | 3.38 | 0.026 ^2^ | 0.04 |
| hsa-miR-320c | 3.08 ± 1.63 | 2.32 (1.70–4.84) | 1.00 ± 0.33 | 0.99 (0.87–1.16) | 2.89 | < 0.001 ^1^ | < 0.001 |
| hsa-miR-663a | 3.27 ± 0.98 | 3.19 (2.61–4.07) | 1.91 ± 1.15 | 1.86 (0.84–2.78) | 2.62 | 0.018 ^2^ | 0.03 |
| hsa-miR-3621 | 2.14 ± 1.02 | 1.95 (1.30–2.97) | 0.97 ± 0.52 | 0.74 (0.58–1.39) | 2.57 | 0.019 ^2^ | 0.03 |
| hsa-miR-6749-5p | 2.05 ± 1.06 | 1.88 (1.13–3.06) | 0.80 ± 0.39 | 0.67 (0.50–0.95) | 2.57 | 0.014 ^2^ | 0.03 |
| hsa-miR-6846-5p | 2.99 ± 1.66 | 2.00 (1.66–4.92) | 0.82 ± 0.31 | 0.79 (0.57–1.11) | 2.45 | < 0.001 ^1^ | < 0.001 |
| hsa-miR-4270 | 2.30 ± 1.25 | 2.34 (1.04–3.45) | 1.17 ± 0.22 | 1.13 (0.94–1.40) | 2.22 | 0.045 ^2^ | 0.05 |
| hsa-miR-486-5p | 2.75 ± 1.93 | 1.79 (1.15–4.32) | 0.85 ± 0.40 | 0.75 (0.51–1.12) | 2.14 | 0.031 ^2^ | 0.05 |
| hsa-miR-6089 | 2.79 ± 1.05 | 2.73 (2.15–3.50) | 1.78 ± 0.42 | 1.70 (1.39–2.04) | 2.00 | 0.039 ^2^ | 0.05 |
| hsa-miR-6752-5p | 3.01 ± 0.87 | 2.82 (2.61–3.89) | 2.05 ± 0.67 | 2.05 (1.66–2.34) | 1.95 | 0.032 ^2^ | 0.05 |
| hsa-miR-4463 | 1.83 ± 1.04 | 1.64 (0.99–2.59) | 0.89 ± 0.34 | 0.94 (0.69–1.17) | 1.75 | 0.049 ^2^ | 0.05 |
| hsa-miR-4651 | 1.96 ± 0.96 | 1.96 (1.20–2.69) | 1.10 ± 0.22 | 1.18 (0.86–1.27) | 1.71 | 0.046 ^2^ | 0.05 |
| hsa-miR-3187-5p | 0.61 ± 0.12 | 0.61 (0.57–0.68) | 1.12 ± 0.42 | 1.15 (1.04–1.39) | -1.52 | 0.001 ^2^ | < 0.01 |
| hsa-miR-3940-3p | 0.49 ± 0.24 | 0.38 (0.33–0.68) | 1.04 ± 0.16 | 0.98 (0.95–1.16) | -1.54 | < 0.001 ^2^ | < 0.001 |
| hsa-miR-6787-5p | 1.51 ± 0.31 | 1.44 (1.25–1.79) | 2.14 ± 0.46 | 2.06 (1.76–2.61) | -1.56 | 0.003 ^2^ | 0.01 |
| hsa-miR-7850-5p | 0.77 ± 0.21 | 0.76 (0.62–0.88) | 1.36 ± 0.39 | 1.45 (1.06–1.56) | -1.59 | 0.001 ^2^ | < 0.01 |
| hsa-miR-4717-3p | 1.05 ± 0.45 | 1.07 (0.74–1.43) | 1.93 ± 0.51 | 1.83 (1.63–2.22) | -1.72 | 0.002 ^2^ | 0.01 |
| hsa-miR-4530 | 1.47 ± 0.20 | 1.43 (1.36–1.54) | 2.26 ± 0.62 | 2.25 (1.61–2.74) | -1.76 | 0.001 ^2^ | < 0.01 |
| hsa-miR-4750-3p | 1.35 ± 0.37 | 1.18 (1.08–1.62) | 2.14 ± 0.62 | 2.18 (1.49–2.68) | -2.00 | 0.005 ^1^ | 0.01 |
| hsa-miR-320e | 6.05 ± 0.58 | 6.02 (5.73–6.47) | 7.13 ± 0.39 | 7.20 (6.73–7.49) | -2.13 | 0.001 ^2^ | < 0.01 |
| hsa-miR-8075 | 6.15 ± 0.75 | 6.22 (5.66–6.62) | 7.19 ± 0.53 | 7.34 (7.05–7.58) | -2.25 | 0.008 ^2^ | 0.02 |
| hsa-miR-6750-5p | 2.49 ± 0.64 | 2.52 (1.98–2.96) | 3.66 ± 1.29 | 3.80 (2.30–4.84) | -2.29 | 0.018 ^2^ | 0.03 |
| hsa-miR-6511b-5p | 2.37 ± 0.90 | 2.44 (1.59–3.11) | 3.72 ± 1.77 | 3.55 (2.67–5.11) | -2.38 | 0.044 ^2^ | 0.05 |
| hsa-miR-4440 | 2.72 ± 0.97 | 2.79 (2.39–3.52) | 4.27 ± 0.80 | 4.37 (3.48–4.83) | -2.66 | 0.004 ^2^ | 0.01 |
| hsa-miR-4706 | 2.85 ± 0.74 | 2.73 (2.32–3.29) | 4.33 ± 0.68 | 4.38 (3.98–4.77) | -3.21 | 0.001 ^2^ | < 0.01 |
| hsa-miR-4668-5p | 2.91 ± 2.27 | 1.68 (1.13–4.58) | 7.00 ± 1.18 | 7.20 (5.95–7.92) | -43.72 | 0.003 ^1^ | 0.01 |
| hsa-miR-3613-3p | 2.71 ± 2.46 | 1.41 (0.84–4.28) | 7.53 ± 1.55 | 7.51 (6.18–8.86) | -70.05 | 0.001 ^1^ | < 0.01 |

Values are presented as mean ± SD and median (interquartile range, IQR). ^1^ Mann–Whitney *U* test; ^2^ Student’s *t*-test. *p*-value < 0.05 and FDR ≤ 0.05 were statistically significant. FC > 1.5 or < -1.5.

T2DM-CAD, type 2 diabetes mellitus with coronary artery disease; FC, fold change; FDR, false discovery rate

**Supplementary Table 3.** Significantly dysregulated miRNAs between the T2DM group and the control group.

| **miRNA** | **T2DM** | | **Controls** | | **FC** | ***p*-Value** | **FDR** |
| --- | --- | --- | --- | --- | --- | --- | --- |
|  | ***n* = 12** | | ***n* = 6** | |  |  |  |
| hsa-miR-6789-5p | 3.69 ± 1.44 | 4.00 (3.05–4.63) | 2.20 ± 1.11 | 1.93 (1.43–2.30) | 5.26 | 0.042 ^1^ | 0.05 |
| hsa-miR-663a | 3.12 ± 1.10 | 2.91 (2.23–3.93) | 1.91 ± 1.15 | 1.86 (0.84–2.78) | 2.10 | 0.045 ^1^ | 0.05 |
| hsa-miR-6794-5p | 2.63 ± 0.54 | 2.64 (2.20–3.17) | 1.93 ± 0.63 | 1.80 (1.76–2.04) | 1.72 | 0.028 ^1^ | 0.05 |
| hsa-miR-6779-5p | 1.68 ± 0.56 | 1.57 (1.28–2.05) | 1.07 ± 0.37 | 0.96 (0.75–1.45) | 1.54 | 0.029 ^1^ | 0.05 |
| hsa-miR-4440 | 2.91 ± 0.95 | 2.92 (2.61–3.32) | 4.27 ± 0.80 | 4.37 (3.48–4.83) | -2.57 | 0.008 ^1^ | 0.05 |

Values are presented as mean ± SD and median (interquartile range, IQR). ^1^ Student’s *t*-test. *p*-value < 0.05 and FDR ≤ 0.05 were statistically significant. FC > 1.5 or < -1.5.

T2DM, type 2 diabetes mellitus; FC, fold change; FDR, false discovery rate

**Supplementary Table 4.** Significantly dysregulated miRNAs between the T2DM-CAD and CAD groups.

| **miRNA** | **T2DM-CAD** | | **CAD** | | **FC** | ***p*-Value** | **FDR** |
| --- | --- | --- | --- | --- | --- | --- | --- |
|  | ***n* = 12** | | ***n* = 8** | |  |  |  |
| hsa-miR-4687-3p | 4.08 ± 1.33 | 4.63 (3.10–5.02) | 2.48 ± 1.15 | 2.58 (1.52–3.41) | 3.91 | 0.013 ^2^ | 0.05 |
| hsa-miR-6724-5p | 5.12 ± 1.01 | 5.34 (4.82–5.67) | 3.53 ± 1.11 | 3.59 (2.44–4.41) | 3.55 | 0.004 ^1^ | 0.03 |
| hsa-miR-1915-3p | 5.95 ± 1.24 | 6.37 (5.69–6.57) | 4.75 ± 1.63 | 5.12 (4.94–5.62) | 2.21 | 0.005 ^1^ | 0.03 |
| hsa-miR-8075 | 6.15 ± 0.75 | 6.22 (5.66–6.62) | 7.43 ± 0.71 | 7.59 (7.16–7.83) | -2.69 | 0.001 ^2^ | 0.02 |

Values are presented as mean ± SD and median (interquartile range, IQR). ^1^ Mann–Whitney *U* test; ^2^ Student’s *t*-test. *p*-value < 0.05 and FDR ≤ 0.05 were statistically significant. FC > 1.5 or < -1.5.

**
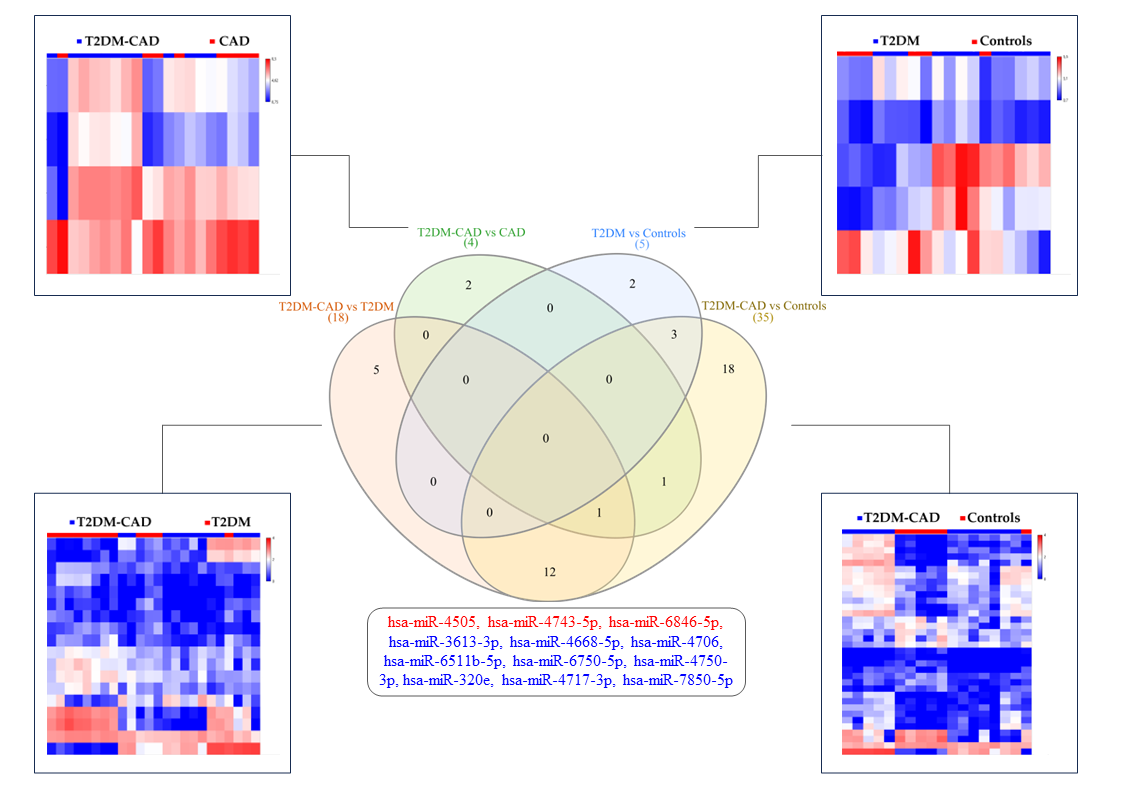
**T2DM-CAD, type 2 diabetes mellitus with coronary artery disease; CAD, coronary artery disease; FC, fold change; FDR, false discovery rate

**Supplementary Figure 1**. Venn diagram showing unique and common DE-miRNAs in T2DM-CAD, T2DM, CAD and control groups. Pairwise comparisons among the study groups in the discovery cohort revealed the intersection of 12 DE-miRNAs between T2DM-CAD vs. T2DM and T2DM-CAD vs. controls (׀FC׀ > 1.5 and FDR ≤ 0.05). The plot was generated using a web-based tool InteractiVenn (https://www.interactivenn.net/, accessed on 18 May 2023) [1]. Heatmaps based on the hierarchical clustering of DE-miRNAs among groups (׀FC׀ > 1.5 and FDR ≤ 0.05). Each column represents a sample group; each row represents a miRNA. The color scale indicates the relative expression of miRNAs, where red shows higher expression and blue lower expression.

DE-miRNA, differentially expressed miRNA; T2DM, type 2 diabetes mellitus; CAD, coronary artery disease; T2DM-CAD, type 2 diabetes mellitus with coronary artery disease; FC, fold change; FDR, false discovery rate


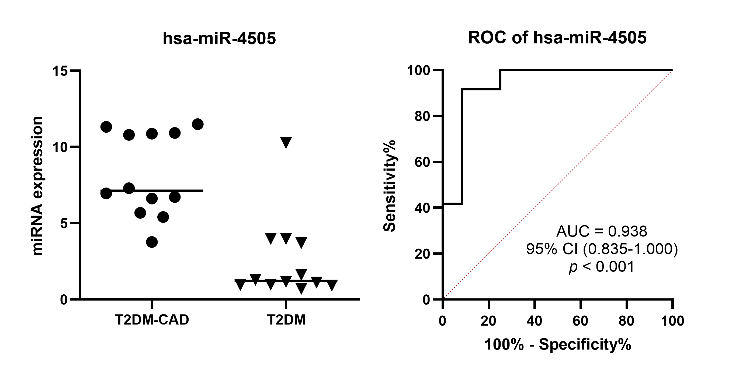

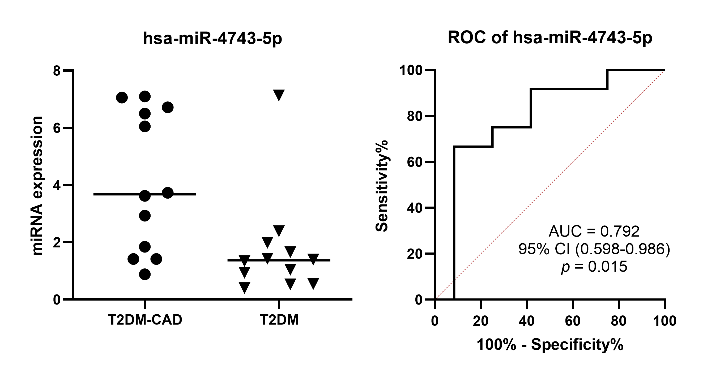

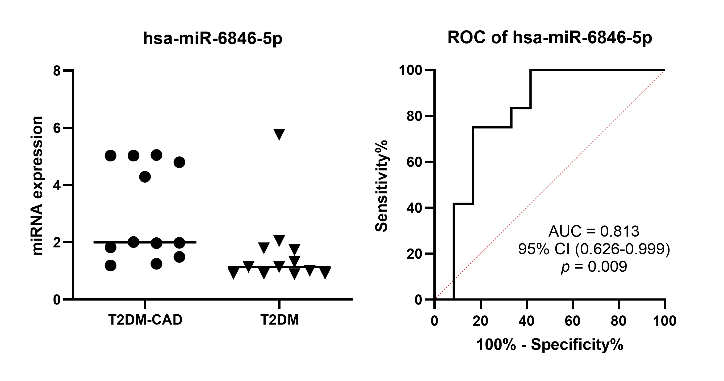

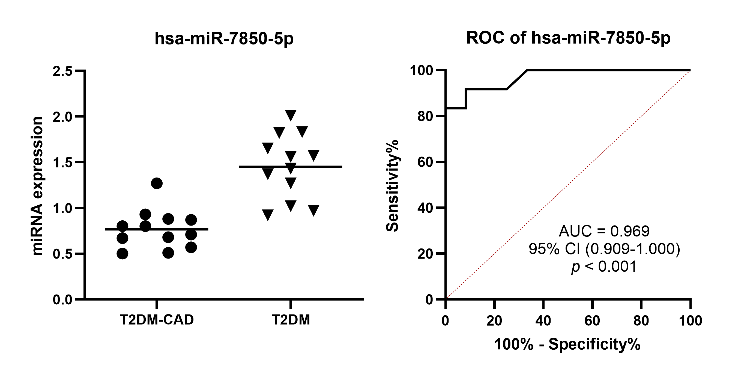

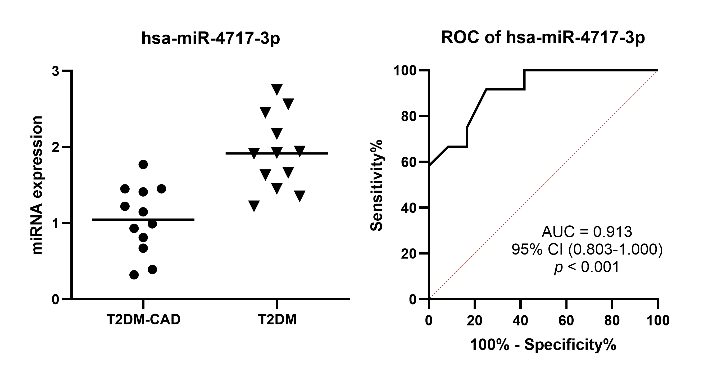

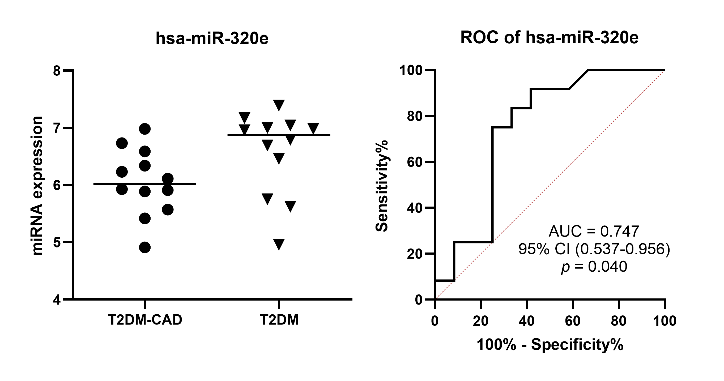

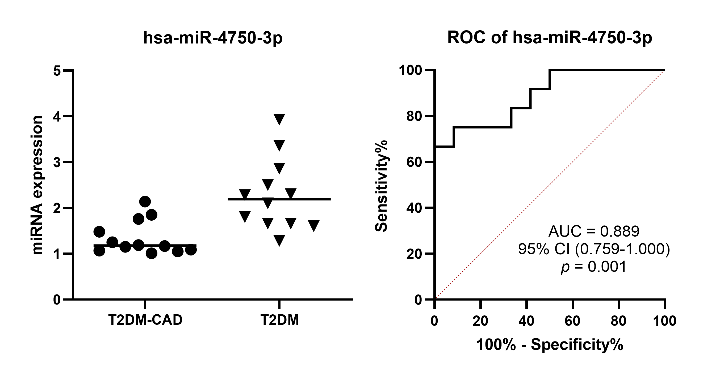

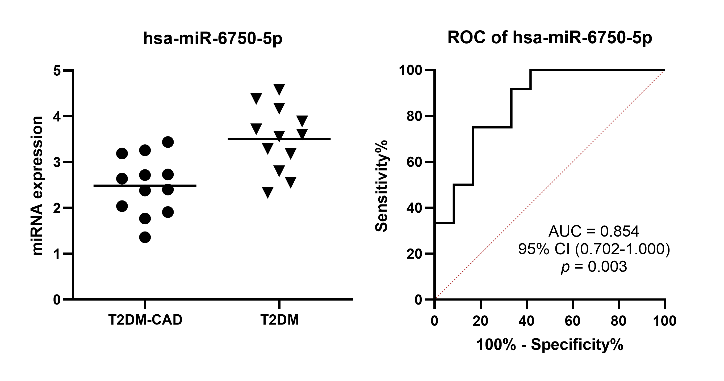


**Supplementary Figure 2.** Differential miRNA expression in the plasma of T2DM-CAD and T2DM patients. Scatter plots show the mean or median expression levels of DE-miRNAs in T2DM-CAD patients compared to T2DM patients from the miRNA profiling study. Differences in miRNA expression levels between groups were compared using Student's *t-*test or Mann-Whitney *U* test, depending on the data distribution. ROC curves and AUC for DE-miRNAs were obtained from the expression level data of miRNA molecules from microarray miRNA profiling. The graphs consist of the AUC value, 95% CI and the level of statistical significance. *p*-value < 0.05 was statistically significant.


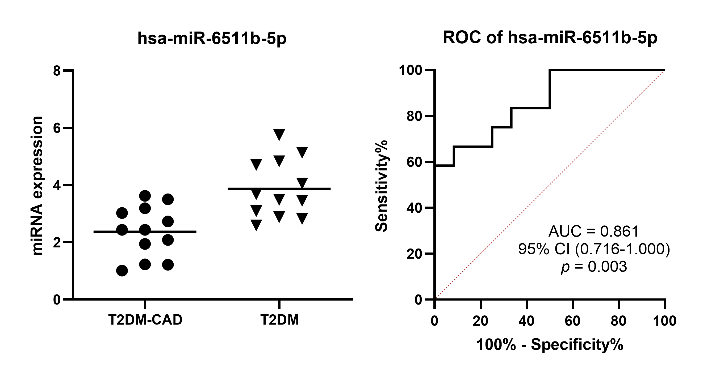

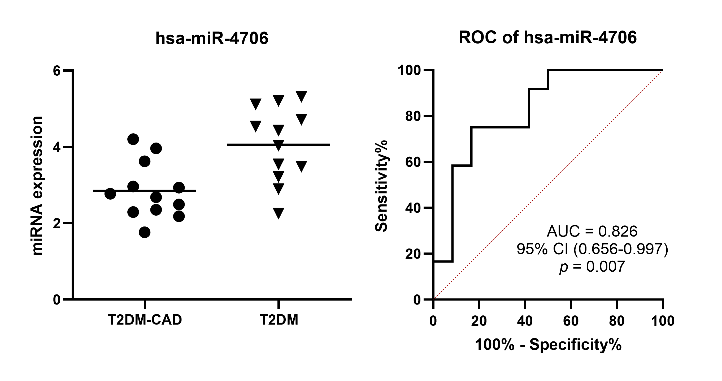

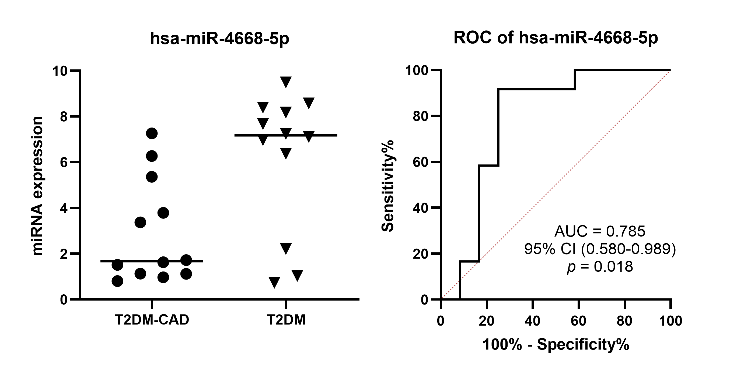

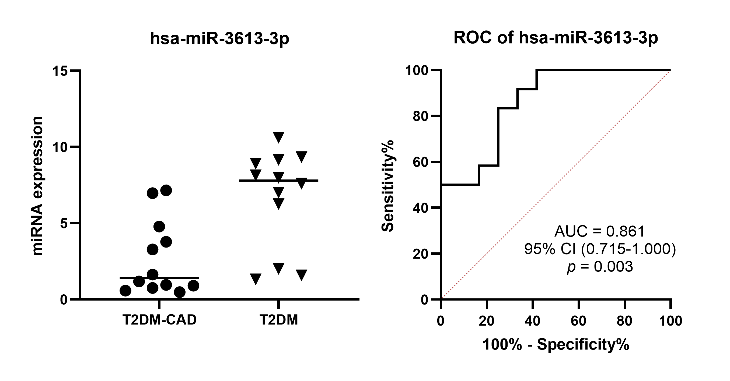


T2DM-CAD, type 2 diabetes mellitus with coronary artery disease; T2DM, type 2 diabetes mellitus; DE-miRNA, differentially expressed miRNA; ROC, receiver operating characteristic; AUC, area under the curve; CI, confidence interval

**Supplementary Table 5.** Tested miRNAs and their target genes.

| **miRNA** | **Targeted genes** | **Number of targets** |
| --- | --- | --- |
| hsa-miR-320e | *ABCA2, ABHD14B, ABI2, AC002316.1, AC011448.1, ACBD6, ACPP, ACVR2B, ADGRL1, AGFG1, AHCYL1, AHNAK, AKAP11, AKAP9, ALDH6A1, ALOX5AP, ALYREF, ANXA11, ANXA6, AP1G1, AP5B1, APAF1, ARF3, ARHGAP11A, ARHGAP17, ARHGAP29, ARID2, ARL10, ARSB, ASXL2, ATE1, ATF7IP, ATN1, ATP13A3, ATP6AP1, ATRX, ATXN2L, AUTS2, AZIN1, B4GALNT4, BAMBI, BAZ2A, BCL2L11, BDP1, BEX1, BLMH, BMF, BTG2, C16orf96, C3, C7orf26, CALU, CAND1, CANX, CAPNS1, CAPRIN2, CAPZA1, CARD10, CASP2, CCDC127, CCDC186, CCDC80, CCND2, CCNT1, CCT7, CDK16, CDK6, CDS1, CDV3, CEP85L, CERS2, CFAP70, CHRNB1, CLASP1, CLIC5, CLN6, CLOCK, CNEP1R1, COL6A1, CPD, CRNKL1, CRTC1, CS, CSNK1A1, CSNK1G1, CYB561D1, DAD1, DBF4, DBNL, DCAF16, DCAF7, DCAF8, DCTN5, DDX19A, DEK, DGAT2, DGCR2, DHX33, DIAPH1, DIPK1B, DMTN, DNAJC13, DPP8, DSP, DTNA, DUSP19, DUSP22, DYNLL2, DYNLRB1, DYRK2, DYRK3, E2F3, ECT2, EEF1D, EGR1, ELK4, ELMOD2, ELOVL1, ERCC1, ESD, EXT1, FAM120A, FAM120B, FBXO17, FBXO5, FDXR, FKBP10, FNBP1L, GFPT1, GGNBP2, GNAS, GOLGB1, GPALPP1, GPRC5B, GTSE1, H2BC21, H6PD, HELB, HERC1, HES6, HIP1, HIPK1, HIVEP3, HNRNPU, HOXA10, HOXA6, HOXC9, HOXD1, HPS5, HSPH1, IDS, IGF1R, IGF2BP3, IL6ST, IPO7, IRF3, IRS4, ISOC2, ITGAV, ITM2C, IVD, JMY, JUN, KIAA0930, KIAA1958, KLHL1, KLHL15, KLHL20, KLHL21, KMT2D, KRIT1, LAPTM4A, LARP4B, LCLAT1, LENG8, LITAF, LMNB2, LNPEP, LRRC8B, LZTS3, MANF, MAP4, MAPK8IP2, MARCKS, MAST4, MBD3, MEAF6, MED6, MED7, MGST3, MID1IP1, MIF4GD, MINK1, MIS18BP1, MMAB, MRPL24, MRPS23, MT-CYB, MT-ND4, MT-ND4L, MYO6, MYRF, NACA, NACC1, NAT10, NAT9, NCAPD2, NCBP3, NCK2, NCOA2, NELFB, NEXMIF, NFAT5, NHLRC2, NOL4L, NPAT, NPM3, NR1H2, NR4A2, NRARP, NRIP1, NSD2, NSD3, NSMF, NUCKS1, NUP98, OAT, OAZ1, OGA, OLFML2A, OSTM1, OXSR1, PABPC1L2A, PABPC4, PAPOLA, PCDHB11, PCDHGA4, PCSK1, PDHA1, PDLIM5, PER3, PGBD4, PHACTR1, PHKG2, PHLPP1, PIGO, PIK3R3, PNRC1, PPP1CC, PPP1R15B, PPP1R37, PPRC1, PPY, PROSER1, PRRG1, PTBP1, PTDSS1, PTPRF, PURA, QSOX1, RAB5B, RABL6, RACGAP1, RAD54L, RAD9A, RANGAP1, RAPGEFL1, RAPH1, RBM14, RBM26, RCE1, RELA, REV3L, RILPL1, RIT1, RLIM, RNASEL, RNF10, RNF11, RNF138, RNF149, RPL22, RPL32, RPS19, RTN4, RUBCN, RXRB, S100A4, SCAF4, SCAMP3, SEC23B, SEC31B, SELENON, SEMA7A, SENP5, SERINC1, SESN3, SETD5, SF3B1, SFPQ, SFXN1, SGCD, SHC1, SIPA1L2, SKAP2, SLC1A3, SLC25A10, SLC25A24, SLC34A2, SLCO5A1, SMARCC1, SOAT1, SOX11, SOX4, SPPL3, SQSTM1, SRCAP, STARD8, STAT6, STX16, SUSD6, SVIL, SYNGR2, SYT11, TASOR2, TDRP, TET3, TFCP2L1, TIMM21, TIMP1, TIMP3, TJP2, TMEM230, TMEM250, TMEM263, TNPO2, TNRC6A, TOMM20, TRAF1, TRAF3IP1, TRAK1, TRAPPC8, TRIM11, TSC22D2, TSPAN3, TUB, TUBB, TUBB4A, TXNL1, UBAP2L, UBE2F, UBE3C, UBLCP1, UBXN7, ULK1, UQCRC1, USP34, VGLL4, WASF2, WDFY2, WDR43, WDR6, WDR77, WNT2, YIPF5, YTHDF3, ZBTB18, ZBTB47, ZDHHC11, ZNF275, ZNF281, ZNF394, ZNF41, ZNF436, ZNF445, ZNF484, ZNF491, ZNF607, ZNF618, ZNF629, ZNF70, ZNF746, ZSWIM8* | 383 |
| hsa-miR-3613-3p | *ABHD13, AC022966.1, ACER2, ACTB, ACTR10, ADAMTS17, ADAMTS9, ADD1, ADH5, ADIPOR2, ADM, AGO2, AGPS, AGTRAP, AHRR, AK4, AKAP1, AKAP2, AKIRIN1, ALG10B, ANAPC16, ANKFY1, ANKRD50, ANTXR2, AP5M1, APH1A, APLF, APOC3l, APOL6, APPBP2, APPL2, AREL1, ARFGEF2, ARFGEF3, ARHGAP12, ARHGAP5, ARID3A, ARIH1, ARRDC3, ASAP2, ASB11, ATE1, ATF7IP2, ATP11B, ATP13A3, ATP5F1A, ATP6V0A2, ATP6V0E1, ATP8A2, ATP9A, ATRNL1, ATXN1L, AVL9, B3GNT7, B4GALT6, BCAT2, BDP1, BIRC5, BMP3, BRD4, BSDC1, BTBD3, BTF3L4, BTG2, BUB3, BVES, C11orf54, C17orf77, C18orf25, C18orf32, C4orf17, C5AR2, C5orf24, C5orf51, C8orf37, C9orf64, CAB39, CALM2, CALU, CAMK2N1, CAMLG, CANT1, CANX, CAPZA1, CARD8, CASC4, CASTOR2, CBLL1, CBX3, CCBE1, CCDC65, CCDC88C, CCNB1IP1, CCND2, CCNF, CCNL1, CCNY, CCS, CD38, CD47, CD59, CD9, CDC5L, CDC73, CDCA2, CDK12, CDK9, CDR2L, CDV3, CEBPB, CELF1, CENPH, CERCAM, CFL2, CFLAR, CGNL1, CHD1, CHD9, CHMP3, CKS2, CLASP1, CLCN6, CLMN, CLN8, CLPB, CLSPN, CMPK1, CNNM3, CNOT4, CNOT6L, CNOT9, COIL, COX18, CPEB2, CPM, CRISPLD2, CRNKL1, CSNK1A1, CTC1, CTNNA3, CTSH, CTTN, CWF19L1, DAZAP1, DBNL, DCAF16, DCDC2, DCP2, DDX6, DEFB105A, DEFB105B, DENND11, DENND4A, DFFA, DHODH, DICER1, DLG4, DLG5, DNA2, DNTTIP2, DPF3, DPP8, DPPA4, DSC3, DUSP1, DYNC1I1, DYNC1LI2, DYNLL2, DYRK1A, DYRK2, ECPAS, EDEM1, EDNRB, EED, EGR1, EHBP1, EIF2S3, EIF3H, EIF4G3, ELMSAN1, ELOA, ENAH, EP300, ERGIC1, ERGIC2, ERO1B, ESCO2, EXOC5, FAM104A, FAM117B, FAM126B, FAM135A, FAM193A, FAM234B, FAM91A1, FANCC, FAT3, FBN2, FBXO11, FBXO25, FBXO31, FBXO47, FBXO48, FCF1, FCHSD2, FDX1, FEM1A, FGF12, FGF5, FKBP5, FKBP9, FNBP1, FOSB, FOXK2, FOXN2, FOXP2, FREM2, FRMD3, FUBP1, FUNDC2, FUT10, FUT11, G3BP1, GABARAPL1, GABPB2, GADD45A, GALK2, GATD3A, GDE1, GDPD1, GEMIN6, GGCX, GIGYF1, GINS2, GJD3, GLP2R, GMCL1, GNAQ, GNPTAB, GOLGA3, GPATCH8, GPBP1L1, GPR37L1, GPR85, GRB10, GRK2, GSDME, GSTCD, GUCD1, HASPIN, HAT1, HAUS3, HCCS, HCFC2, HECTD1, HEPH, HHIP, HIF1A, HINFP, HINT1, HIPK1, HIPK3, HIVEP1, HK2, HMGB2, HMGCS1, HNRNPD, HNRNPDL, HNRNPR, HNRNPU, HOOK3, HRH4, HSPA1B, HSPA4, HTT, HUWE1, HYPK, IFI44L, IFIT3 IFNLR1, IGF1R, IGFBP5, IGSF11, IGSF9B, INPP4A, INPP4B, INTS7, INTU, IPMK, ISLR2, ITGB8, ITM2A, JAG1, JUNB, KCNAB1, KCNB1, KCNJ11, KCNK5, KCNMB3, KCNN3, KCTD2, KDM1B, KIF13A, KIF2A, KLF7, KLHDC8A, KLHL11, KMT2C, LAMTOR1 LAPTM4A, LARP4B, LCP1, LDLR, LDLRAD4, LENG8, LIG4, LIMA1, LIN7C, LINC00598, LMBR1, LMNB1, LMNB2, LPCAT3, LPGAT1, LRIF1, LRPAP1, LRRC58, LRTOMT, LSG1, LSM11, LSM8, LYZ, MACC1, MAFK, MALT1, MAP3K3, MAP7, MAPK8, MAST4, MAT1A, MAVS, MBNL3, MBOAT2, MCM10, MCM4, MCM9, MCTS1, MDM2, MED28, METTL7A, MEX3C, MFSD9, MGAT3, MICA, MIDN, MKNK2, MLXIP, MMGT1, MOB1A, MOB1B, MORF4L1, MPRIP, MPZL1, MSL3, MT1G, MTX3, MXRA7, MYBL1, MYOCD, NACC1, NCBP3, NCOA1, NCOA4, NCOR2, NDRG4, NFYA, NGDN, NINJ1, NIPAL1, NKAPD1, NOP53, NPC1, NPLOC4, NPM3, NR2F2, NRAS, NRIP2, NT5DC3, NUDCD3, NUDT21, NUP160, NXN, OCIAD2, OGFOD1, OLR1, OR9Q1, ORC1, ORC4, OTUD1, PAFAH1B1, PAFAH1B2, PAK3, PAK4, PALM2AKAP2, PAQR7, PARD6B, PARP15, PARVB, PCNX2, PDE3A, PDF, PDIA5, PDK3, PDP2, PDZRN4, PEG3, PELI1, PFDN2, PFKP, PFN1, PGM2, PHAX, PHF14, PHF7, PHKG2, PIK3C2B, PIM3, PLA2G2C, PLA2G4D, PLAGL2, PLCXD1, PLEKHA3, PLEKHG3, PLK2, PLXDC1, PLXNC1, PNO1, PNRC1, POGZ, POLK, POLM, POLR2D, POLR2M, PPFIBP1, PPIB, PPIP5K2, PPM1A, PPP1R12C, PPP4R3A, PRKAR1A, PRKCI, PROSER3, PRPF19, PRPF8, PRR14L, PRRG3, PRXL2C, PSMB2, PSMD11, PSMG1, PSPC1, PTBP3, PTCD2, PTCHD1, PTMA, PURA, QTRT2, RAB18, RAB3GAP2, RABGEF1, RAD51L3-RFFL, RAN, RANBP10, RAP1B, RAPH1, RASSF2, RBM14, RBM26, RCAN3, REL, RELCH, RERE, RESF1, RFFL, RHOA, RIC1, RNF103-CHMP3, RNF115, RNF2, RNF20, RNF207, RNF38, RPF2, RPL10A, RPL32, RPL3L, RPP40, RPS16, RPS28, RRAGC, S100PBP, S1PR2, S1PR3, SAMD15, SAMD8, SCN1A, SCYL3, SECISBP2L, SEH1L, SENP5, SESN3, SFT2D2, SFXN1, SGMS2, SGO2, SGSM2, SH2B3, SH3BP2, SHMT1, SHOX2, SIKE1, SINHCAF, SIX1, SKA1, SKIL, SKP2, SLBP, SLC14A1, SLC19A1, SLC24A4, SLC25A12, SLC25A37, SLC30A1, SLC35C2, SLC35D1, SLC35E4, SLC36A4, SLC38A2, SLC39A9, SLFN5, SLK, SMAD2, SMARCA5, SMG1, SMIM7, SMS, SNAP29, SNIP1, SOD2, SOS1, SOWAHB, SOX4, SOX6, SPATS2, SPC24, SPECC1, SPIC, SPIDR, SPPL3, SPTSSA, SQSTM1, SRFBP1, SRP19, SRPX2, SRSF2, STAG2, STARD4, STAT2, STEAP4, STK17B, STMP1, STRIP2, STXBP2, SUGP2, SUN1, SV2C, SYAP1, SYDE2, SYNM, TACC1, TAF13, TANGO2, TAOK1, TARDBP, TBL1XR1, TBPL1, TBX20, TCAF1, TCF7L2, TENT4B, TENT5D, TET2, TET3, TGIF1, TGIF2, TGOLN2, THOC2, TIPRL, TLN1, TM4SF20, TM4SF5, TMC5, TMED10, TMED2, TMED8, TMEM127, TMEM154, TMEM158, TMEM170A, TMEM170B, TMEM196, TMEM220, TMEM33, TMEM44, TMEM65, TMF1, TMSB4X, TMTC1, TNFAIP8, TNFRSF10B, TNFRSF13C, TNFRSF9, TNPO1, TNRC6A, TNRC6B, TP53INP1, TP73, TRA2B, TRAF3IP1, TRAM1, TRAM2, TRAPPC10, TRAPPC6B, TRIM66, TRIO, TRMT112, TRPS1, TSC22D3, TSPEAR-AS2, TTPAL, TUBB2A, TULP4, TVP23C, TXNIP, UBE2D3, UBE2E1, UBE2Z, UBE3A, UBN2, UBXN7, UGCG, UHRF1BP1L, USF2, USF3, USP6NL, USP7, UTP20, VGLL4, VIM, VPS37A, VPS50, VTI1A, WASF3, WDFY1, WDR12, WDR13, WDR33, WDR76, WEE1, WIPF2, WNK3, WNT2B, WT1, XRN1, YY1, ZBTB14, ZBTB3, ZBTB33, ZBTB39, ZBTB41, ZER1, ZFAND6, ZFC3H1, ZFP36L1, ZFP91, ZFY, ZFYVE26, ZMAT3, ZNF124, ZNF136, ZNF157, ZNF226, ZNF236, ZNF257, ZNF275, ZNF28, ZNF282, ZNF284, ZNF317, ZNF394, ZNF451, ZNF484, ZNF487, ZNF514, ZNF518B, ZNF549, ZNF552, ZNF562, ZNF568, ZNF574, ZNF581, ZNF582, ZNF619, ZNF641, ZNF654, ZNF664, ZNF678, ZNF860, ZNF99, ZNFX1, ZWINT* | 721 |
| hsa-miR-4505 | *ACVRL1, ADAM17, ADAR, AKAP1, ANKRD13B, ARHGDIA, ARL2, ARL8A, ARSK, ASB6, ATL3, BCL2L1, BCL9L, C10orf67, C9orf62, CABP4, CACNA1C, CBX6, CBX8, CD4, CDC37, CDK2, CDKN1A, CELF1, CENPO, CEP126, CEP135, CEP57L1, CLU, COX6B1, CRIPT, CSK, DLG5, DMRT2, DNAJB9, ECE1, ELP2, FBXL19, FBXW8, FGF19, FGFR1OP, FOXJ2, FOXK1, FSCN1, FURIN, GDE1, GJA5, GRM1, GTF3C1, GUK1, H2AC18, H2AC19, HECA, HIC2, HOOK3, IBA57, IL7R, ILK, IMP4, INMT, IRAK3, ITGA3, KCNH2, KDM6B, KHSRP, KIAA0895L, KLHDC8A, KRT8, L2HGDH, LACTB, LEAP2, LINC00346, LLGL1, MACF1, MAGI3, MAP1S, MAP2K7, MAPK8IP3, MDK, MED17, MED28, MEMO1, METTL6, MICOS10, MKI67, MLX, MMP17, MNT, MOB1B, MSH5, MVK, MYADM, NAA10, NCKAP5L, NDEL1, NECTIN1, NEURL1, NF2, NIBAN2, NTSR1, ORC4, PABPN1, PAK4, PARD6B, PARP2, PARVB, PCP4L1, PCSK6, PDE6B, PEX2, PKM, PLIN3, PNMA8B, PODXL, POLR2E, POPDC2, PRKCA, PSMD3, PTDSS2, PTEN, PTGIS, PTPA, R3HDM4, RAB5B, RAP1GAP2, RDH10, REXO1, RGS6, RNF126, RNF40, RPL37A, RTBDN, RTL8A, SEC22C, SEPTIN14, SFT2D2, SHISA2, SIGLEC9, SLC10A3, SLC12A7, SLC25A6, SLC27A1, SLC2A1, SLC35C2, SLIT1, SMCR8, STAC, STX4, SUGT1, SUPT16H, SYTL3, TAF1, TAF8, TBCCD1, TFAP4, TGOLN2, THOC5, TIAL1, TMEM184A, TMEM248, TMEM43, TMEM63C, TRAF7, TRAM2, TRIM28, TSKU, U2AF2, UBN2, ULBP3, WDR73, XPNPEP3, YES1, ZC3H7B, ZCCHC8, ZNF24, ZNF284, ZNF321P, ZNF551, ZNF562, ZNF703, ZNF774* | 181 |
| hsa-miR-4668-5p | *ABCC9, ACADSB, ACAP2, ACER2, ACTR10, ADH5, AGTRAP, AK4, AKAP11, ALYREF, ANP32E, AP1M1, AP1S1, AP3M2, AP5S1, AR, ARGLU1, ARIH1, ARL5B, ATAT1, ATPAF1, AVL9, AXIN2, B3GNT7, B4GALT1, BAZ2A, BCL10, BSCL2, C17orf75, C5orf51, CACNA2D2, CALM1, CARD8, CBY3, CCDC117, CCDC58, CCND2, CCNF, CD46, CDC5L, CDCA3, CDKN1A, CDKN2AIPNL, CDR2L, CENPA, CFAP65, CFL1, CHD3, CISD1, CLDN4, CLSPN, CNKSR3, CNN3, CNNM4, CNOT6L, CPM, CPT1B, CRCP, CRISPLD2, CWF19L1, CYB561, CYP4F11, DCLK2, DENND10, DLC1, DLG4, DLG5, DNTTIP2, DPM2, DPP6, DPP8, DR1, DRAM2, DUSP4, DYRK4, E2F2, E2F3, EDIL3, EEF1AKMT2, EFNA1, EIF4A3, ELL, ENSA, EPB41L4B, EPHB4, EPN1, ETV3, EXT1, FAM104A, FAM117B, FAM118A, FAM174B, FARSA, FBN2, FCHSD2, FECH, FEM1A, FGF12, FGF2, FHL2, FICD, FLCN, FOXA1, FRK, FUT11, G6PC, GABARAP, GAN, GBP6, GEN1, GJD3, GK5, GNG3, GNPTAB, GPR183, GPR37L1, GPR61, GRK2, GSTCD, GTPBP2, HACD2, HAPLN1, HAVCR1, HCCS, HDGF, HDLBP, HECTD1, HMGB1, HMGN2, HOXA13, HS3ST5, IGF1R, IGSF11, INCENP, INTS7, INTU, IPPK, ITGA3, ITGB8, ITM2C, KCNB1, KCNE4, KCNK6, KCTD11, KDELR1, KHSRP, KIAA0895L, KLHL12, KLHL18, KLHL28, KMT2A, KNSTRN, KRTAP5-4, KRTAP6-1, LACC1, LATS2, LCOR, LDB1, LDHD, LIMD1, LIX1, LMAN1, LMAN2, LMNB2, LRIF1, LRRC8B, LYN, LYZ, MAOB, MARC1, MAT2A, MAX, MAZ, MC2R, MCOLN3, MCRIP2, MDM4, MED30, MEF2C, MELK, METTL1, METTL8, MKNK2, MLF2, MMS22L, MOCS3, MORF4L1, MORF4L2, MPEG1, MRI1, MTX3, MYC, MYO1C, NAA15, NACC2, NAV2, NCBP3, NCEH1, NEGR1, NEK2, NF2, NFYA, NPM3, NRIP2, NXT2, OCRL, OGFOD1, OGT, ORAI1, PAFAH1B2, PAQR7, PARD6B, PARVB, PCBD1, PCNT, PDE4D, PDF, PDGFRA, PDHA1, PDPK1, PDRG1, PEX7, PHACTR2, PHF12, PHKG2, PIGS, PIP4K2C, PKM, PLXNA3, PMPCA, PNRC1, POLR2D, POU4F1, PPARGC1A, PPIA, PPIL1, PPM1K, PPP1CB, PRDM2, PRIM1, PRR3, PSMD11, PTP4A1, PXMP4, QSER1, RAB30, RAB6A, RABGAP1, RAD51B, RAD54L2, RAP1GDS1, RAP2B, RASGEF1B, RASL11A, RBM12B, RBM28, RCC2, REST, RFTN2, RFX3, RGMB, RMDN1, RNF44, RORA, RPL24, RRP7A, RTTN, SALL3, SCD, SDC1, SEC63, SEH1L, SEL1L, SEPTIN6, SESTD1, SET, SFT2D2, SH3BP2, SH3BP5L, SH3RF1, SHOC2, SHOX2, SIAH3, SIN3A, SINHCAF, SLC19A1, SLC24A3, SLC24A4, SLC25A21, SLC25A36, SLC25A6, SLC30A4, SLC35C2, SLC35E2A, SLC44A1, SLC7A5, SMAD4, SMIM19, SNRPD1, SNX33, SOD2, SOX6, SP1,SP4, SPART, SPATS2, SPIC, SREBF1, SRFBP1, SRPX2, STK17B, STK4, STMN1, STMP1, STRIP2, SVOP, TAB2, TACO1, TAF12, TAF13, TAF1D, TAPBP, TBC1D2B, TBL1XR1, TBRG1, TENT4B, TERB2, TET3, TFDP3, TGFBR1, TGIF1, TM4SF20, TM4SF5, TMBIM6, TMED10, TMEM164, TMEM167A, TMEM220, TMEM65, TNFRSF13C, TNPO1, TNRC6A, TOR1AIP2, TP73, TRAPPC8, TRIM14, TRIM21, TRPM6, TRPS1, TSC1, TSTD2, TTLL5, TUFT1, TUSC1, UBE2H, UBE3C, UBE4B, UBXN7, UCP1, UHRF1BP1L, UNC45B, UNG, USF1, USF3, USP15, USP2, USP45, USP6NL, VANGL2, VEGFC, VIM, VPS35, VPS50, VSTM5, WASF3, WEE1, WIPF2, WNK3, WTIP, XKR4, XRCC2, ZBTB10, ZBTB3, ZFP91, ZMYM1, ZMYM6, ZNF132, ZNF229, ZNF236, ZNF284, ZNF354B, ZNF460, ZNF557, ZNF568, ZNF579, ZNF581, ZNF608, ZNF619, ZNF713, ZNF730, ZNF746, ZYG11B* | 402 |
| hsa-miR-4706 | *ABHD12, AKT1S1, ANKRD45, ARHGAP31, ARHGAP39, ARL8A, ASB6, ATXN1L, BARHL1, BTF3L4, C12orf49, C1D, C3orf36, CACNA1A, CALR, CASZ1, CC2D1B, CHERP, CLCN7, CLU, CTC1, CTTN, DIRAS1, DISC1, DUSP14, EFHD2, F2R, FNDC3B, FSCN1, GATAD2A, GIPC1, GNAI2, GPR20, GRB2, H2AX, HCFC1, HIC1, HOXD11, HOXD3, INKA2, KDM6B, KMT2D, LHFPL3, LTBP4, MAFK, MKNK2, MLLT1, MRPL44, NECTIN1, NFAT5, NFIX, OTUB1, OTUD4, PAK4, PDE4C, PDZD4, PER1, PEX26, PPTC7, RAB17, RAB5C, REG3A, RGS5, SBF1, SBK1, SCAMP4, SEMA3F, SENP2, SH3BGRL3, SIGLEC12, SLC10A7, SLC47A1, SLC7A5, SLC9A3, SPECC1L, SRCIN1, TACC3, TGFA, THSD4, THY1, TMEM184B, TOR4A, TULP1, VGLL4, YIPF2, ZCCHC3, ZNF516, ZNF562, ZNF787* | 89 |
| hsa-miR-4717-3p | *ABCA6, ADAM28, ALG1, ANGPT4, ARNT, ATG2A, ATP5MD, BSDC1, BTC, CDKN1A, CELF2, CEP57L1, CIDEC, CNOT6, COX15, CRISPLD2, DAZAP2, DSTN, DYRK2, EN2, EXOSC6, FHOD1, FOXR2, G3BP1, HAPLN1, HIPK3, KLF6, LRRC58, METTL24, MTAP, MTRNR2L4, MYLK, NACC2, NAIF1, NLRC3, NUP98, PCNP, PDE7B, PLCG2, PLXNA2, PNMA8A, PRRG4, QDPR, RCC2, RNF168, RPP14, RSL24D1, SEPTIN9, SERINC3, SH3PXD2A, SLC16A10, SLC7A1, SPECC1, SRSF2, STUM, SUSD1, SYNGR2, TFDP2, TGIF1, TNFAIP8, TPK1, TPM3, TWNK, TXNIP, UBN2, UGDH, VAV3, WRN, YWHAQ, ZBED3, ZDHHC20, ZFAND4, ZFP36L2, ZNF100, ZNF131, ZNF485, ZNF543, ZNF578* | 78 |
| hsa-miR-4743-5p | *ALKBH1, AP5Z1, ARC, ARL2, ATL3, ATP8B1, BAZ2B, BCL10, BCR, C16orf58, C1QTNF6, CBX5, CBX8, CCDC71L, CERS1, CHAC1, CHCHD4, COL8A1, CYP20A1, DLL4, HSPA6, IFFO2, IGSF8, JUNB, KCNE4, LGALS3BP, LINC01551, MAP2K3, MOB3A, NAT9, NDRG1, NXN, PEX26, PIGR, PLCD1, RBM22, RBM38, RPL24, RTN4RL2, SEMA3E, SERF1A, SERF1B, SH3BP4, SHANK3, SLC1A2, SLC25A11, SNRPD1, SNRPD3, SPINDOC, SPPL2A, SREBF1, TEDC1, TRAF3IP1, TRIP10, U2AF2, ULBP3, ULK2, USP22, ZNF70* | 59 |
| hsa-miR-4750-3p | *ACTN4, ANKRD27, ARID1A, BRWD1, CCDC9B, CDK1, CDK6, CREBRF, DNAJB5, FAM83G, FLG2, GPER1, IFI44L, KCNN1, KIF1B, KRT80, MED28, MEX3A, MKNK2, NFATC4, PDE4C, PHB2, PLAAT3, PLEKHM2, PTMA, RGS11, RORA, RTL6, SERPINH1, SH2D5, STK40, SYTL4, TP53INP1, TP73, VAT1, WEE2, XPC, ZBTB25* | 38 |
| hsa-miR-6511b-5p | *ADAM28, AGO2, AGO3, AKAP11, ATOX1, BARX1, BCAP31, BHMT2, BSN, CANX, CAPN1, CFAP73, CLDN4, COX19, CSK, CYCS, DCTN6, ERG28, ETV7, FGFR1OP, GALNS, GATA2, GBA2, GDF5OS, GJB1, GLG1, GOSR2, GPR155, GPRIN3, GRK7, GSTO2, HAS2, HCN4, HDGF, HIC2, HNRNPA3, HOXC8, HSD17B12, HSPB6, HSPG2, ICOSLG, IGFBP4, IPP, KLF3, LDLR, LLGL1, LMNB2, LRRC58, MECP2, MED13, MEIS1, MIXL1, MSRB2, MYPN, NAP1L4, NFASC, NGFR, NIF3L1, NLRC5, NUDT8, NUP98, PERP, PITPNC1, POGK, POLQ, PUS3, RAB3B, RAB9A, RABL2A, RABL2B, RBPJ, RNF11, RNF19B, RPL30, RUFY2, SEC22C, SEMA5A, SFTPB, SIX6, SLC30A2, SLC35F5, SNRPD3, SNX29, SPATA5, SRGAP1, STAG3L3, STX17, SVIP, TENT5B, TMEM134, TMEM184B, TMEM245, TNFAIP8L3, TRIM72, TRIM73, TRIM74, UBE2G2, UBXN2A, UHMK1, VCL, XKR7, YIPF4, ZDHHC14, ZNF17, ZNF317, ZNF385A, ZNF708, ZSCAN25* | 108 |
| hsa-miR-6750-5p | *ACTR2, AGO2, AP2M1, ATXN1L, BHLHE40, BICRAL, BMP8A, BMPR1A, C19orf12, C3orf80, CAMKK2, CASTOR2, CST9, DLX2, DVL3, EIF4E3, EREG, ERGIC2, FKBP15, FKBP4, GABPB1, GABRQ, GBA2, GTF2IRD2, HIRIP3, HLA-B, HLA-C, HMGN5, HSPA1B, INCENP, LDHD, M6PR, MLF2, MPPE1, MYH2, NAT9, NCOA2, NPEPPS, NPTX1, NPTX2, NRGN, NUFIP2, NUP85, PHB2, PHF12, POLR2E, PPIA, PPP1CB, PPP2CA, QSER1, RAP1A, RBM38, REL, RNLS, RPRD2, RPS6KA5, SLC25A36, SMOC1, SNX17, SOBP, SOCS7, STK35, THUMPD3, TM9SF4, TMEM119, TUBB, TUBB2A, UBA1, UCHL5, UHMK1, ZFAND2B, ZNF500* | 72 |
| hsa-miR-6846-5p | *ABCC5, ACVRL1, ANKLE1, APEX1, ARGFX, ARHGAP18, ARPC1B, ATP6AP1, BCL7A, CALM3, CALR, CBARP, CBX6, CCDC71L, CD55, CLU, CNBP, COLGALT1, COX10, CS, CTXN1, DCAKD, DDX39B, DPM2, E2F1, EFNB1, EIF5AL1, ENTPD5, ERCC1, FAM151B, FARSA, FBRS, FEM1A, FNDC3B, FSCN1, G6PC3, G6PD, GDE1, GNB2, GPAT4, GPATCH3, HDGF, HMCN1, HMGA1, HS6ST1, KHSRP, MAPK8IP2, MARK2, MEN1, MICB, MIDN, MRNIP, MSN, MTHFSD, MYH11, NACC1, NACC2, NCKAP5L, NFIC, PDE4A, POLR2F, PPP1R11, PRELP, PRR12, PRSS8, RAPGEF1, RNF185, ROBO4, RPL28, RRP7A, SARS1, SBF1, SERF1A, SERF1B, SHISA2, SIK1, SIX5, SLC10A3, SLC10A7, SLC35E2A, SMOX, SOCS7, SRCIN1, STEAP3, STMN3, STX1A, TBC1D28, TFAP4, THRA, THSD4, TJAP1, TMEM109, TPD52L3, TRAF3, TXLNA, UBE2D4, VPS37C, WBP2, WDR45B, ZBTB7A, ZC3H7B* | 101 |
| hsa-miR-7850-5p | *ABAT, ADM, ARSB, ATG10, ATP5MC1, ATRX, AZF1, C21orf91, CALU, CCDC9B, CCL16, CCS, CENPN, CHCHD5, CHMP3, CMKLR1, CNDP1, COX6B1, DDX19A, DEFB105A, DEFB105B, DTHD1, DUSP18, EEF2, EIF1AX, EMP2, EN2, FAM184B, FAM83G, FNDC3A, FRK, GATA6, GATAD1, GRK3, GSTCD, H2AC11, H3C14, H3C15, HAND2, HNRNPC, HNRNPU, HTR7, IKZF2, KCNB1, KCNJ2, KCNJ6, KRT8, LLPH, LSM12, MAP3K13, MDM2, MESD, MGAT5, MTRF1L, MYO10, MYO18A, MYOCD, NKAIN1, NPRL3, OGT, ONECUT3, OTUD7B, P2RY10, PARD3, PDZD8, PIM1, PITX1, POU6F2, PPM1K, PRDM10, PRICKLE4, PRKX, PTPN14, PVR, RBMXL1, RCC2, RND2, RNF103-CHMP3, RNF165, SAMD12, SLC16A1, SRRM4, TEX22, TIAF1, TIMM10B, TMEM67, TRABD2A, TTYH1, UBXN2A, VAPA, WNT2B, WWC2, XKR6, YAE1, YWHAB, ZIC5, ZNF224, ZNF275, ZNF384, ZNF550* | 100 |

Based on the STRING database, we obtained a total of 341 and 1,991 experimentally validated target genes of the up-regulated and down-regulated miRNAs, respectively. For the three up-regulated miRNAs, hsa-miR-4505 was found to potentially target the most genes, with the number of 181, while for the nine down-regulated miRNAs, hsa-miR-3613-3p possessed the most targets, which number is 721.

**Supplementary Table 6.** Functional enrichment analysis of the six top-scored clusters for up-regulated miRNA target genes in T2DM-CAD.

| **Cluster** | **Category** | **Term** | ***p*-Value** | **Adjusted  *p*-Value** | **Genes** |
| --- | --- | --- | --- | --- | --- |
| I | GO:BP | mRNA processing | 1.34 x 10^-9^ | 6.39 x 10^-6^ | *PABPN1, U2AF2, DDX39B, KHSRP, THOC5, ZCCHC8* |
|  | GO:BP | RNA splicing | 4.98 x 10^-10^ | 6.39 x 10^-6^ | *PABPN1, U2AF2, DDX39B, KHSRP, THOC5, ZCCHC8* |
|  | GO:CC | Ribonucleoprotein complex | 1.0 x 10^-6^ | 0.002 | *PABPN1, U2AF2, TIAL1, DDX39B, ZCCHC8* |
|  | GO:MF | RNA binding | 3.07 x 10^-8^ | 9.93 x 10^-5^ | *PABPN1, U2AF2, TIAL1, DDX39B, KHSRP, THOC5, ZCCHC8* |
|  | Reactome | mRNA 3’-end processing | 2.77 x 10^-9^ | 6.02 x 10^-6^ | *PABPN1, U2AF2, DDX39B, THOC5* |
| II | GO:CC | Chromosome, telomeric region | 1.15 x 10^-5^ | 0.020 | *ERCC1, CDK2, ORC4* |
|  | KEGG | Cell cycle | 8.32 x 10^-6^ | 0.003 | *CDK2, E2F1, ORC4* |
|  | Reactome | G2 Phase | 2.3 x 10^-6^ | 0.005 | *CDK2, E2F1* |
|  | Reactome | Cell cycle | 3.92 x 10^-5^ | 0.007 | *CDK2, E2F1, MSH5, ORC4* |
| III | GO:CC | Anchoring junction | 2.57 x 10^-6^ | 0.004 | *ITGA3, HMCN1, NF2, ILK, PARVB* |
|  | KEGG | Hippo signaling pathway - multiple species | 4.43 x 10^-5^ | 0.012 | *NF2, MOB1B* |
|  | KEGG | Focal adhesion | 3.62 x 10^-5^ | 0.012 | *ITGA3, ILK, PARVB* |
|  | Reactome | Cell-extracellular matrix interactions | 2.08 x 10^-5^ | 0.045 | *ILK, PARVB* |
| IV | GO:CC | Phagophore assembly site membrane | 1.2 x 10^-5^ | 0.020 | *WDR45B, ULK2* |
| V | GO:BP | Translation | 1.83 x 10^-6^ | 0.024 | *RPL24, RPL37A, EIF5AL1, RPL28* |
|  | GO:CC | Cytosolic large ribosomal subunit | 5.17 x 10^-7^ | 8.8 x 10^-4^ | *RPL24, RPL37A, RPL28* |
|  | GO:MF | Structural constituent of ribosome | 1.09 x 10^-5^ | 0.035 | *RPL24, RPL37A, RPL28* |
|  | KEGG | Ribosome | 6.04 x 10^-6^ | 0.002 | *RPL24, RPL37A, RPL28* |
|  | Reactome | Peptide chain elongation | 1.86 x 10^-6^ | 0.004 | *RPL24, RPL37A, RPL28* |
| VI | GO:BP | Regulation of ion transmembrane transport | 8.88 x 10^-9^ | 6.34 x 10^-6^ | *KCNH2, CACNA1C, KCNE4, CALM3, GJA5* |
|  | GO:BP | Regulation of cation transmembrane transport | 4.47 x 10^-7^ | 2.0 x 10^-4^ | *KCNH2, CACNA1C, KCNE4, CALM3* |
|  | GO:CC | Cation channel complex | 1.32 x 10^-5^ | 0.011 | *KCNH2, CACNA1C, CALM3* |
|  | GO:CC | Calcium channel complex | 1.1 x 10^-4^ | 0.027 | *CACNA1C, CALM3* |
|  | GO:MF | Voltage-gated cation channel activity | 3.55 x 10^-6^ | 0.005 | *KCNH2, CACNA1C, KCNE4* |

The most enriched T2DM-CAD-related terms from the three Gene Ontology (GO) subcategories (Biological Process - GO:BP, Cellular Component - GO:CC, Molecular Function - GO:MF), Kyoto Encyclopedia of Genes and Genomes (KEGG) and/or Reactome pathways are listed for each cluster. *p*-value and adjusted *p*-value < 0.05 were statistically significant.

T2DM-CAD, type 2 diabetes mellitus with coronary artery disease

**Supplementary Table 7.** Functional enrichment analysis of the six top-scored clusters for down-regulated miRNA target genes in T2DM-CAD.

| **Cluster** | **Category** | **Term** | ***p*-Value** | **Adjusted  *p*-Value** | **Genes** |
| --- | --- | --- | --- | --- | --- |
| VII | GO:BP | Cell cycle | 5.26 x 10^-42^ | 6.76 x 10^-38^ | *FBXO5, KNSTRN, MCM4, CLASP1, DBF4, CDK6, PLK2, CDK16, CENPH, SKA1, MELK, BIRC5, ESCO2, MIS18BP1, CLSPN, NCAPD2, GSG2, TACC3, CDCA2, CENPA, SGOL2, PRIM1, NEK2, BUB3, ORC1, RAD54L, ZWINT, CKS2, ECT2 INCENP, CDK1, CCNF, WEE2, KIF2A, WEE1, RACGAP1, GTSE1, MCM10, ORC4, CDCA3, SPC24, ANAPC16* |
|  | GO:BP | Cellular response to stress | 2.5 x 10^-4^ | 0.025 | *GINS2, MCM4, POLQ, PLK2, MELK, ESCO2, CLSPN, MCM9, RAD54L, ECT2, CDK1, GTSE1, MCM10* |
|  | GO:MF | Protein serine/threonine kinase activity | 1.1 x 10^-4^ | 0.019 | *CDK6, PLK2, CDK16, MELK, GSG2, NEK2, CDK1* |
|  | KEGG | Cell cycle | 2.86 x 10^-11^ | 9.59 x 10^-9^ | *MCM4, DBF4, CDK6, BUB3, ORC1, CDK1, WEE2, WEE1, ORC4* |
|  | Reactome | Cell Cycle | 3.14 x 10^-30^ | 6.83 x 10^-27^ | *FBXO5, GINS2, MCM4, CLASP1, DBF4, CDK6, CENPH, SKA1, ESCO2, MIS18BP1, CLSPN, NCAPD2, CENPA, SGOL2, PRIM1, NEK2, BUB3, ORC1, ZWINT, CENPN, INCENP, CDK1, KIF2A, WEE1, GTSE1, MCM10, ORC4, SPC24, ANAPC16* |
| VIII | GO:BP | Positive regulation of nitrogen compound metabolic process | 5.75 x 10^-9^ | 5.68 x 10^-6^ | *USF2, NR1H2, KDM6B, SRCAP, EP300, BRD4, STAT6, KMT2D, CEBPB, SP1, HMGB1, GATA2, DUSP19, USF1, DR1, TMSB4X, NCOA1* |
|  | GO:BP | Positive regulation of metabolic process | 9.48 x 10^-9^ | 8.12 x 10^-6^ | *USF2, NR1H2, KDM6B, SRCAP, EP300, BRD4, STAT6, KMT2D, CEBPB, SP1, HMGB1, GATA2, DUSP19, ACTB, USF1, DR1, TMSB4X, NCOA1* |
|  | GO:BP | Positive regulation of blood vessel endothelial cell migration | 8.07 x 10^-7^ | 2.5 x 10^-4^ | *SP1, HMGB1, GATA2, TMSB4X* |
|  | GO:BP | Response to oxygen-containing compound | 5.93 x 10^-5^ | 0.008 | *KDM6B, STAT6, CEBPB, SP1, HMGB1, ACTB, USF1, NCOA1, CFL1* |
|  | GO:BP | Positive regulation of vascular endothelial cell proliferation | 2.2 x 10^-4^ | 0.019 | *SP1, HMGB1* |
|  | GO:BP | Regulation of NIK/NF-kappaB signaling | 3.5 x 10^-4^ | 0.027 | *EP300, HMGB1, TMSB4X* |
|  | GO:BP | Lipid homeostasis | 7.2 x 10^-4^ | 0.045 | *USF2, NR1H2, USF1* |
|  | GO:BP | Leukocyte activation | 7.5 x 10^-4^ | 0.046 | *EP300, STAT6, CEBPB, TUBB, HMGB1, PPIA* |
|  | Reactome | Platelet degranulation | 1.83 x 10^-5^ | 0.004 | *PFN1, TMSB4X, PPIA, CFL1* |
|  | Reactome | NR1H3 & NR1H2 regulate gene expression linked to cholesterol transport and efflux | 1.55 x 10^-5^ | 0.004 | *NR1H2, EP300, NCOA1* |
| IX | GO:BP | Translation | 2.32 x 10^-20^ | 2.98 x 10^-16^ | *RPS16, RPL3L, PDF, RPL22, RPL10A, RPL24, EEF1D, RPL32, RPL30, EIF3H, NACA, RPS19, RPS28* |
|  | GO:CC | Focal adhesion | 1.42 x 10^-5^ | 0.002 | *RPS16, RPL22, RPL10A, RPL30, RPS19* |
|  | GO:MF | Structural constituent of ribosome | 2.2 x 10^-15^ | 7.14 x 10^-12^ | *RPS16, RPL3L, RPL22, RPL10A, RPL24, RPL32, RPL30, RPS19, RPS28* |
|  | KEGG | Ribosome | 3.86 x 10^-16^ | 1.3 x 10^-13^ | *RPS16, RPL3L, RPL22, RPL10A, RPL24, RPL32, RPL30, RPS19, RPS28* |
|  | Reactome | Eukaryotic Translation Elongation | 6.58 x 10^-20^ | 1.43 x 10^-16^ | *RPS16, RPL3L, RPL22, RPL10A, RPL24, EEF1D, RPL32, RPL30, RPS19, RPS28* |
| X | GO:BP | DNA repair | 1.5 x 10^-19^ | 1.93 x 10^-15^ | *POLK, UNG, HELB, FANCC, WRN, GEN1, DNA2, XRCC2, POLM, RAD51B, ENSP00000466834, LIG4* |
|  | GO:MF | Ion binding | 3.4 x 10^-4^ | 0.027 | *POLK, HELB, WRN, GEN1, DNA2, XRCC2, POLM, RAD51B, ENSP00000466834, LIG4* |
|  | KEGG | Homologous recombination | 1.99 x 10^-6^ | 6.7 x 10^-4^ | *XRCC2, RAD51B, ENSP00000466834* |
|  | Reactome | DNA Repair | 7.35 x 10^-17^ | 1.6 x 10^-13^ | *POLK, UNG, FANCC, WRN, GEN1, DNA2, XRCC2, POLM, RAD51B, LIG4* |
| XI | GO:BP | RNA biosynthetic process | 3.52 x 10^-14^ | 4.53 x 10^-10^ | *TBPL1, CCNT1, ELL, POLR2D, POLR2M, NELFB, TAF13, CDK9, NFIX, POLR2E* |
|  | GO:BP | Nitrogen compound metabolic process | 3.43 x 10^-6^ | 0.001 | *TBPL1, CCNT1, ELL, POLR2D, ASB6, POLR2M, NELFB, TAF13, CDK9, NFIX, CDK12, POLR2E* |
|  | GO:CC | Transferase complex, transferring phosphorus-containing groups | 2.72 x 10^-13^ | 4.61 x 10^-10^ | *TBPL1, CCNT1, POLR2D, POLR2M, TAF13, CDK9, CDK12, POLR2E* |
|  | GO:MF | DNA-directed 5-3 RNA polymerase activity | 2.3 x 10^-6^ | 0.007 | *POLR2D, POLR2M, POLR2E* |
|  | Reactome | TP53 Regulates Transcription of DNA Repair Genes | 5.26 x 10^-15^ | 1.14 x 10^-11^ | *CCNT1, ELL, POLR2D, NELFB, CDK9, CDK12, POLR2E* |
|  | Reactome | SMAD2/SMAD3:SMAD4 heterotrimer regulates transcription | 1.9 x 10^-4^ | 0.009 | *CCNT1, CDK9* |
| XII | GO:CC | Cytoplasmic stress granule | 4.56 x 10^-8^ | 7.75 x 10^-5^ | *FAM195A, PABPC4, ATXN2L, DDX6* |
|  | GO:CC | Cytoplasmic ribonucleoprotein granule | 5.86 x 10^-8^ | 7.75 x 10^-5^ | *FAM195A, PABPC4, DCP2, ATXN2L, DDX6* |
|  | KEGG | RNA degradation | 5.06 x 10^-8^ | 1.7 x 10^-5^ | *PABPC4, PABPC1L2A, DCP2, DDX6* |

The most enriched T2DM-CAD-related terms from the three Gene Ontology (GO) subcategories (Biological Process - GO:BP, Cellular Component - GO:CC, Molecular Function - GO:MF), Kyoto Encyclopedia of Genes and Genomes (KEGG) and/or Reactome pathways are listed for each cluster. *p*-value and adjusted *p*-value < 0.05 were statistically significant.

T2DM-CAD, type 2 diabetes mellitus with coronary artery disease

**Supplementary Table 8.** Summary of ROC analysis for testing the diagnostic performance of miRNAs as biomarkers for T2DM-CAD.

| **DE-miRNAs** | **AUC** | **95% CI** | ***p*-Value** | **Cut-off point** | **Se [%]** | **Sp [%]** |
| --- | --- | --- | --- | --- | --- | --- |
| hsa-miR-4505 | 0.876 | 0.791–0.960 | < 0.001 | 0.22 | 80.00 | 83.33 |
| hsa-miR-4743-5p | 0.860 | 0.768–0.952 | < 0.001 | 0.43 | 86.67 | 76.67 |
| hsa-miR-4750-3p | 0.833 | 0.726–0.939 | < 0.001 | -0.54 | 73.33 | 86.67 |

ROC, receiver operating characteristic; AUC, area under the curve; CI, confidence interval; DE-miRNA, differentially expressed miRNA; Se, sensitivity; Sp, specificity

**Supplementary Table 9.** Summary of basic parameters and standard quality measures of miRNA-based models.

| **Models** | **Variables** | **AUC** | **95% CI** | ***p*-Value** | **Se [%]** | **Sp [%]** | **PPV [%]** | **NPV [%]** | **Intercept** | **Coefficients** |
| --- | --- | --- | --- | --- | --- | --- | --- | --- | --- | --- |
| Model 1 | x_1_ = hsa-miR-4505 | 0.913 | 0.846–0.981 | < 0.0001 | 90.00 | 76.67 | 79.41 | 88.46 | -1.839 | x_1_ = 2.518 |
|  | x_2_ = hsa-miR-4743-5p |  |  |  |  |  |  |  |  | x_2_ = 2.244 |
| Model 2 | x_1_ = hsa-miR-4505 | 0.930 | 0.867–0.993 | < 0.0001 | 86.70 | 80.00 | 81.30 | 85.70 | -2.301 | x_1_ = 3.826 |
|  | x_2_ = hsa-miR-4750-3p |  |  |  |  |  |  |  |  | x_2_ = -3.604 |
| Model 3 | x_1_ = hsa-miR-4743-5p | 0.950 | 0.896–1.000 | < 0.0001 | 96.67 | 86.67 | 87.88 | 96.30 | -4.466 | x_1_ = 4.799 |
|  | x_2_ = hsa-miR-4750-3p |  |  |  |  |  |  |  |  | x_2_ = -4.629 |
| Model 4 | x_1_ = hsa-miR-4505 | 0.959 | 0.914–1.000 | < 0.0001 | 100.00 | 86.67 | 88.24 | 100.00 | -3.945 | x_1_ = 2.546 |
|  | x_2_ = hsa-miR-4743-5p |  |  |  |  |  |  |  |  | x_2_ = 3.389 |
|  | x_3_ = hsa-miR-4750-3p |  |  |  |  |  |  |  |  | x_3_ = -4.208 |

ROC, receiver operating characteristic; AUC, area under the curve; CI, confidence interval; Se, sensitivity; Sp, specificity; PPV, positive predictive value; NPV, negative predictive value

**References**

1. Heberle H, Meirelles GV, da Silva FR, Telles GP, Minghim R. InteractiVenn: a web-based tool for the analysis of sets through Venn diagrams. BMC Bioinformatics. 2015;16:169.
